# Comparative genomics of the core and accessory genomes of 48 *Sinorhizobium *strains comprising five genospecies

**DOI:** 10.1186/gb-2013-14-2-r17

**Published:** 2013-02-20

**Authors:** Masayuki Sugawara, Brendan Epstein, Brian D Badgley, Tatsuya Unno, Lei Xu, Jennifer Reese, Prasad Gyaneshwar, Roxanne Denny, Joann Mudge, Arvind K Bharti, Andrew D Farmer, Gregory D May, Jimmy E Woodward, Claudine Médigue, David Vallenet, Aurélie Lajus, Zoé Rouy, Betsy Martinez-Vaz, Peter Tiffin, Nevin D Young, Michael J Sadowsky

**Affiliations:** 1 BioTechnology Institute, 1479 Gortner Ave, 140 Gortner Labs, University of Minnesota, St Paul, MN 55108, USA; 2 Department of Plant Biology, 250 Biological Sciences, 1445 Gortner Ave, University of Minnesota, St Paul, MN 55108, USA; 3 Department of Biological Sciences, 3209 N. Maryland Ave, University of Wisconsin-Milwaukee, Milwaukee, WI 53211, USA; 4 National Center for Genome Resources, 2935 Rodeo Park Drive East, Santa Fe, NM 87505, USA; 5 CNRS, UMR8030 & UEVE, Université d'Evry & CEA/DSV/IG/Genoscope, Laboratoire d'Analyses Bioinformatiques pour la Génomique et le Métabolisme, Centre National de Séquençage, 2 rue Gaston Crémieux CP5706 91057, Evry cedex, France; 6 Department of Biology, MS-B1807, 1536 Hewitt Avenue, Hamline University, St Paul, MN 55104, USA; 7 Department of Plant Pathology, 495 Borlaug Hall, 1991 Upper Buford Circle, University of Minnesota, St Paul, MN 55108, USA; 8 Department of Soil, Water, & Climate, 491 Borlaug Hall, 1991 Upper Buford Circle, University of Minnesota, St Paul, MN 55108, USA

## Abstract

**Background:**

The sinorhizobia are amongst the most well studied members of nitrogen-fixing root nodule bacteria and contribute substantial amounts of fixed nitrogen to the biosphere. While the alfalfa symbiont *Sinorhizobium meliloti *RM 1021 was one of the first rhizobial strains to be completely sequenced, little information is available about the genomes of this large and diverse species group.

**Results:**

Here we report the draft assembly and annotation of 48 strains of *Sinorhizobium *comprising five genospecies. While *S. meliloti *and *S. medicae *are taxonomically related, they displayed different nodulation patterns on diverse *Medicago *host plants, and have differences in gene content, including those involved in conjugation and organic sulfur utilization. Genes involved in Nod factor and polysaccharide biosynthesis, denitrification and type III, IV, and VI secretion systems also vary within and between species. Symbiotic phenotyping and mutational analyses indicated that some type IV secretion genes are symbiosis-related and involved in nitrogen fixation efficiency. Moreover, there is a correlation between the presence of type IV secretion systems, heme biosynthesis and microaerobic denitrification genes, and symbiotic efficiency.

**Conclusions:**

Our results suggest that each *Sinorhizobium *strain uses a slightly different strategy to obtain maximum compatibility with a host plant. This large genome data set provides useful information to better understand the functional features of five *Sinorhizobium *species, especially compatibility in legume-*Sinorhizobium *interactions. The diversity of genes present in the accessory genomes of members of this genus indicates that each bacterium has adopted slightly different strategies to interact with diverse plant genera and soil environments.

## Background

The rhizobia are symbiotic nitrogen-fixing bacteria that form root and/or stem nodules on leguminous plants. Within nodules rhizobia convert atmospheric dinitrogen (N_2_) gas into ammonia, resulting in improved plant growth and productivity, even under N-limiting environmental conditions. These bacteria are among the largest fixers of atmospheric N_2 _gas in the biosphere and account for the deposition of nearly 100 to 195 teragrams per year. The effective use of biological nitrogen fixation via application of rhizobia leads to sustainable cropping systems with a net positive impact on the environment [1]. Most currently recognized legume-nodulating bacteria belong to the α-proteobacteria and are members of the genera *Allorhizobium*, *Azorhizobium*, *Mesorhizobium*, *Rhizobium*, *Sinorhizobium *(renamed *Ensifer*), or *Bradyrhizobium *[2,3]. Recently, some members of the β- and γ-proteobacteria have also been shown to nodulate legume plants [4].

Members of the genus *Sinorhizobium *are among the most studied and first sequenced rhizobia. *Sinorhizobium meliloti *(previously *Rhizobium meliloti *and now *Ensifer meliloti*) and its close relative *Sinorhizobium medicae *induce the formation of root nodules on *Medicago *species, including *Medicago truncatula *and *Medicago sativa *(alfalfa) [5]. In contrast, *Sinorhizobium saheli *and *Sinorhizobium terangae *form root and stem nodules with woody leguminous plants, such as *Sesbania *or *Acacia *[6], while *Sinorhizobium fredii *has a very wide host range, nodulating more than 79 plant genera representing all three subfamilies of the family Leguminosae. Although whole genome sequences of some strains of *S. meliloti*, *S. medicae *and *S*. *fredii *have been published [7-12], and many of their genetic features have been well characterized, only a limited number of strains of each species have been well characterized at the genome level. Recently, Tian *et al. *[12] reported the comparative genomics of nine strains of *S*. *fredii *and Baily *et al. *[13] reported the population genomics of 12 *S. medicae *strains analyzed using Roche 454 technology. Moreover, only limited comparative genomics studies among each species exist and there are no reports of genomic feature of other species of *Sinorhizobium*, including the important symbionts of *Sesbania*/*Acacia*.

Most rhizobial nodulation genes (*nod*, *noe*, and *nol*) are involved in the synthesis of host-specific lipochitinoligosaccharide (LCO) Nod factors essential for initial infection [14]. Bacterial genes encoding various polysaccharides, cyclic β-glucans, and type III, IV and VI secretion systems are also involved in symbiosis and host specificity [15-17]. Most of the genes involved in symbiosis are located on large self-transmissible megaplasmids (pSym), or within large genomic symbiotic islands [18]. The megaplasmid pSymA, which has the most symbiosis-related genes in *S. meliloti*, is a more variable replicon than the chromosome or pSymB in this bacterium [10]. Symbiosis-related genes have previously been shown to be highly variable among rhizobial species and strains [10,19] and acquired by via horizontal gene- and plasmid-transfer events. This results in gene replacement and rearrangements leading to genome plasticity [18] and recombination [12] and, ultimately, specificity of symbiotic interactions with their legume hosts. This suggests that gene content in *Sinorhizobium *strains should vary among strains or species and these alterations could influence their symbiotic phenotype on a host plant. However, few comparative genomic studies have focused on gene content or symbiotic function of multiple strains within or between species of sinorhizobia.

Here we describe the assembly and annotation of the whole genomes of 48 strains of *Sinorhizobium *described previously [20], with primary focus on *S. meliloti *and *S. medicae*. While we previously examined 44 of these genomes to characterize population diversity at the single nucleotide level and to determine the forces driving adaptive evolution, our overall goal here was to compare gene content among a large number of strains within a single sinorhizobial species. This was done to better understand functional features in each species and to identify symbiosis-associated genes contributing to symbiotic phenotypes as part of large genome-wide association, SNP, and Hapmap studies [20-22]. Here we show: 1) the genomic features of each *Sinorhizobium *species; 2) the differences in gene content between *S. meliloti *and the taxonomically and symbiotically related species *S*. *medicae*; and 3) the differences among strains and species in genes involved in Nod factor biosynthesis, polysaccharide biosynthesis, protein secretion systems, anaerobic denitrification, and organic sulfur utilization. We also report pair-wise analyses of symbiotic associations of these 46 *S. meliloti *and *S*. *medicae *strains with 27 diverse *M*. *truncatula *genotypes to better understand the relationship of symbiotic phenotype with bacterial genome content.

## Results and discussion

### General features of *Sinorhizobium *genomes

**Figure 1:**
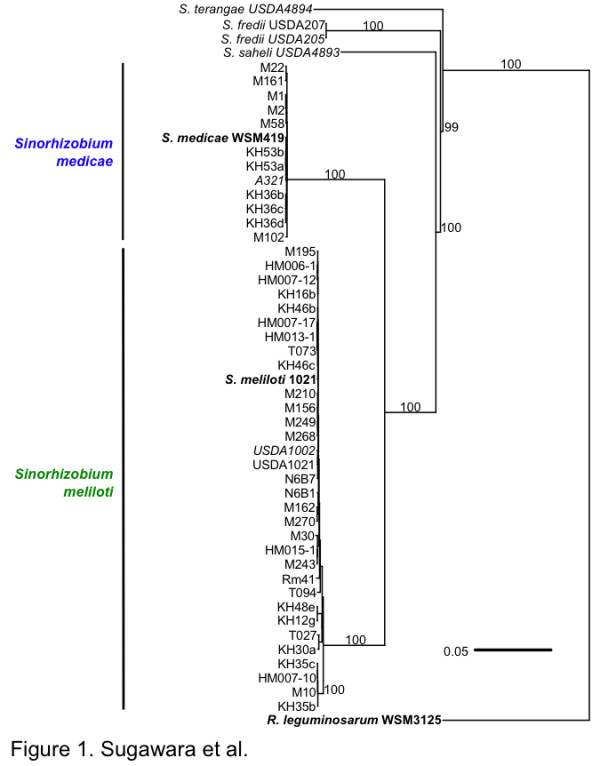
**Neighbor-joining tree based on concatenated sequences for 645 protein coding genes**. Strains that were sequenced in other studies are in bold font and type strains are in italic font. Support for splits was assessed using 1,000 bootstraps, and splits with less than 60% support were collapsed to polytomies. For clarity, the bootstrap values are only shown for the deep branches. Bar indicates number of substitutions per site.

**Figure 2:**
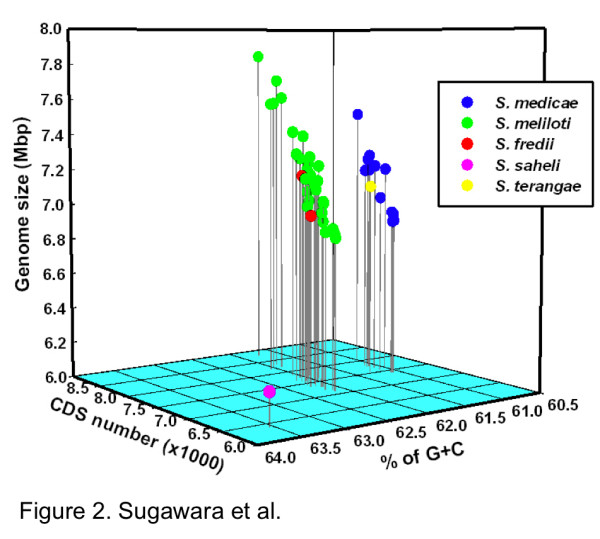
**Three-dimensional plots of genome size, coding sequence number and GC content of the 48 *Sinorhizobium *strains sequenced**.

Annotated draft genome assemblies of 48 *Sinorhizobium *strains comprising five genospecies - *S. meliloti*, *S. medicae*, *S*. *fredii*, *S*. *saheli *and *S*. *terangae *- are presented here (Table S1 in Additional file 1). These assemblies were generated from raw reads used previously to call SNPs in a population genetics analysis [20]. A phylogenetic tree based on 645 protein-coding genes (Figure 1) showed that *S. meliloti *and *S. medicae *are more closely related to each other than to three other species included in this study. A phylogenetic tree based on the 16S rRNA gene sequence (Figure S1 in Additional file 2) was similar to that shown in Figure 1, but the bootstrap values did not support the nodes to the extent of the tree made from protein coding genes. Genome characteristics are summarized in Table S2 in Additional file 1. Total genome sizes varied between species and strains and ranged from 6.2 to 7.8 Mb. The number of predicted protein coding sequences (CDSs; 6,436 to 8,858), and mean mole percentage G+C content (61.0 to 63.5%) also varied among sequenced genomes (Figure 2; Table S2 in Additional file 1). The mean percentage G+C content of *S. meliloti *strains (61.8 to 62.2% for all 32 strains) was greater than those seen in *S. medicae *(60.9 to 61.1% for all 12 strains) (Figure 2). Genome sizes and CDS counts varied greatly among strains in the same species. While *S. meliloti *M270 had the largest genome size (7.8 Mb) and number of CDSs (8,858) among all the tested strains, the genome of *S. saheli *USDA 4893 had the smallest genome size (approximately 6.2 Mb) and highest G+C content (63.5%). The genomes of *S. fredii *and *S. terangae *were similar to those of *S. meliloti *or *S. medicae*, respectively (Figure 2; Table S2 in Additional file 1). Recently, Tian *et al. *[12] reported a comparative analysis of nine *S. fredii *genomes and found that the average genome size was approximately 6.6 Mb, and consisted of a large number of accessory genes likely acquired by horizontal gene transfer. This is similar to what we report here. All of the strains examined contained from two to five plasmids as determined by Eckhart gel electrophoresis.

### Gene contents in *Sinorhizobium *strains

**Figure 3:**
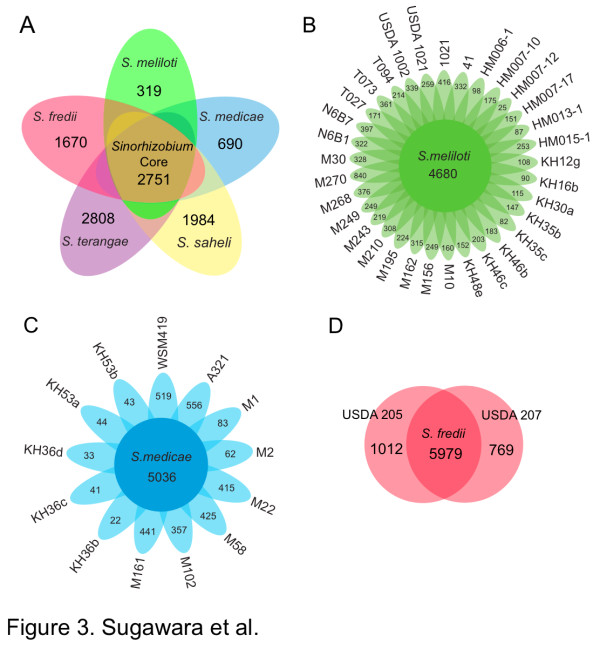
**The pan-genome of *Sinorhizobium***. The flower plots and Venn diagrams illustrate the number of shared and specific (accessory) genes based on clusters of orthologs. **(a) **Flower plot showing numbers of species-specific genes commonly found in each genome of each species (in the petals), and *Sinorhizobium *core orthologous gene number (in the center). **(b) **Flower plots showing numbers of unique orthologous genes in each *S. meliloti *strain (in the petals), and *S. meliloti *core orthologous gene number (in the center). **(c) **Flower plots showing numbers of unique orthologous gene in each *S. medicae *strain (in the petals), and *S. medicae *core orthologous gene number (in the center). **(d) **Venn diagram showing numbers of unique orthologous genes in each *S. fredii *strain, and *S. fredii *core orthologous gene number.

To understand the pan-genome of *Sinorhizobium *more deeply, 380,371 protein CDSs obtained from the 48 newly sequenced genomes plus two reference strains (*S. meliloti *1021 and *S. medicae *WSM419) were clustered using the CD-HIT algorithm with a 70% sequence identity cut-off. A total of 34,150 clusters were identified, and of these, 2,751 orthologs (8%) were identified in all 50 strains as the *Sinorhizobium *core genome (Figure 3a). The remaining variable 31,399 clusters were defined as the *Sinorhizobium *accessory genome. Species-specific genes were identified among the five tested species (Figure 3a).

Species core orthologous genes and strain-specific unique genes within a given *Sinorhizobium *species were examined in 33, 13, and 2 strains of *S. meliloti*, *S. medicae*, and *S. fredii*, respectively (Figure 3b-d). In the *S. meliloti *strains, 21,118 orthologous genes were identified from 33 strains, and of these, 4,680 orthologs were present in all tested *S. meliloti *strains as the species core genome (Figure 3b). The number of unique genes in each *S. meliloti *strain varied from 25 to 840 (Figure 3b). *S. meliloti *strain M270 had the largest genome (7.8 Mb) and the largest number (840) of unique genes. The M270 genome uniquely contained well-correlated regions of the nopaline-type plasmid, pTiC58, found in the plant pathogen *Agrobacterium tumefaciens *C58. This included complete sets of *trb *genes (encoding type IV secretion system proteins involved in conjugal transfer) and nopaline utilization genes (*noc*).

### Functional features of the core and accessory sinorhizobial genomes

**Figure 4:**
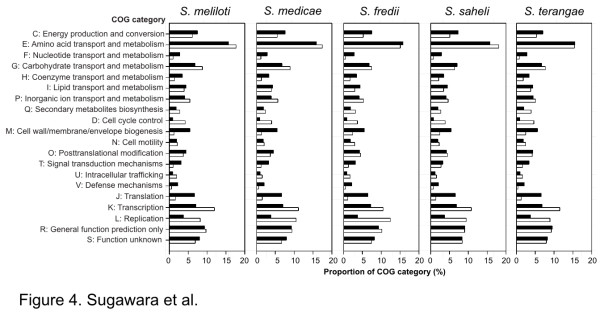
**Distribution of orthologous genes based on COG category in each *Sinorhizobium *species**. The percentages of orthologous genes assigned by COG category in the core genome (black bars) and the accessory genome (white bars) are shown. Only orthologous genes assigned by COGnitor were used for analysis.

To define possible differences in functions encoded by the core and/or accessory genome in each species group, the proportion of proteins in each COG (Clusters of Orthologous Groups) category was plotted versus COG function. Figure 4 shows that the core-genomes in each *Sinorhizobium *species group were commonly enriched in COG categories C, F, H, M, J, and V relative to those seen in the accessory genomes. In contrast, accessory genomes were commonly enriched in COG categories Q, D, K, and L relative to those of the core genome. There was no major difference in COG category proportion between *S. meliloti *and *S. medicae*, but the abundances of genes in category G (carbohydrate transport and metabolism) in the accessory genomes were greater in both of these species strains compared to those seen in other sinorhizobia.

### Functional differences between *S. meliloti *and *S. medicae *

While *S. meliloti *and *S. medicae *are taxonomically related (Figure 1) with somewhat similar host ranges [5], 421 out of 4,680 *S. meliloti *core orthlogous genes were not found in the tested 13 strains of *S. medicae*. Similarly, 396 out of 5,036 *S. medicae *core orthologous genes were not found in the 33 tested strains of *S. meliloti*. Selected *S. meliloti- *or *S. medicae*-specific genes in each species are shown in Table 1 and all species-specific genes are presented in Tables S3 and S4 in Additional file 1. These results show that genes involved in conjugation, C1 metabolism, detoxification, and cellular process were specifically identified in the core genomes of each species. In addition, *S. meliloti *specifically possesses genes encoding a nitrate transporter (*nrtABC*), a nitrogen regulatory protein (*ntrR*), and a succinoglycan biosynthetic gene (*exoI_1_*). In contrast, *S*. *medicae *species specifically contain many arylsulfatase genes (Figure S2 in Additional file 2) associated with transporter genes. Of particular interest is the prevalence of genes involved in organic sulfur utilization in *S. medicae*, which are also present and expressed in *Bradyrhizobium japonicum *when in symbiosis with soybean [23]. This is likely to be of functional importance as organic sulfur in the form of sulfur esters and sulfonates constitute approximately 95% of the total sulfur in aerobic soils [24].

**Table 1:** Selected *S. meliloti*- or *S*. *medicae*-specific genes among both species^a^

Species	Gene ID^b^	Gene name	Function
**Conjugation**			
*S. meliloti*	SMa0929	*traG*	Conjugal transfer coupling protein TraG
*S. meliloti*	SMa0934	*traA_1_*	Conjugal transfer protein TraA1
*S. meliloti*	SMa1302	*virB_11_*	Type IV secretion protein VirB11
*S. meliloti*	SMa1303	*virB_10_*	Type IV secretion protein VirB10
*S. meliloti*	SMa1306	*virB_9_*	Type IV secretion protein VirB9
*S. meliloti*	SMa1308	*virB_8_*	Type IV secretion protein VirB8
*S. meliloti*	SMa1311	*virB_6_*	Type IV secretion protein VirB6
*S. meliloti*	SMa1313	*virB_5_*	Type IV secretion protein VirB5
*S. meliloti*	SMa1315	*virB_4_*	Type IV secretion protein VirB4
*S. meliloti*	SMa1318	*virB_3_*	Type IV secretion protein VirB3
*S. meliloti*	SMa1319	*virB_2_*	Type IV secretion protein VirB2
*S. meliloti*	SMa1321	*virB_1_*	Type IV secretion protein VirB1
*S. meliloti*	SMa1323	*rctA*	Negative transcriptional regulator of *vir *genes
*S. medicae*	Smed_5050	*traD*	Conjugal transfer TraD family protein
*S. medicae*	Smed_5051	*traC*	Conjugal transfer protein TraC
*S. medicae*	Smed_5375	*traI*	Acyl-homoserine-lactone synthase
*S. medicae*	Smed_5377	*trbC*	Conjugal transfer protein TrbC
*S. medicae*	Smed_5387	*traR*	Transcriptional activator protein TraR
*S. medicae*	Smed_5388	*traM*	Transcriptional repressor TraM
*S. medicae*	Smed_5391	*traB*	Conjugal transfer protein TraB
**Nitrogen metabolism**			
*S. meliloti*	SMa0228	*gdhA*	Glutamate dehydrogenase
*S. meliloti*	SMa0581	*nrtC*	Nitrate transport ATP binding protein
*S. meliloti*	SMa0583	*nrtB*	Nitrate ABC transporter permease
*S. meliloti*	SMa0585	*nrtA*	Nitrate ABC transporter substrate-binding protein
*S. meliloti*	SMa0981	*ntrR_2_*	NtrR2 transcription regulator
*S. meliloti*	SMc01521	*ntrR_1_*	Nitrogen regulatory protein
*S. medicae*	Smed_1742	*fnrN*	Nitrogen fixation regulatory protein
**Organic sulfur utilization**			
*S. medicae*	Smed_1128	*ssuB-*like	Aliphatic sulfonates import ATP-binding protein
*S. medicae*	Smed_1129	*ssuA-*like	Aliphatic sulfonates family ABC transporter, periplasmic ligand-binding protein
*S. medicae*	Smed_1130	*atsA-*like	Arylsulfatase
*S. medicae*	Smed_3146	*atsA-*like	Arylsulfatase
*S. medicae*	Smed_3147	*ssuA*	Aliphatic sulfonates family ABC transporter, periplasmic ligand-binding protein
*S. medicae*	Smed_3148	*ssuB*	Sulfonate ABC transporter, ATP-binding protein
*S. medicae*	Smed_3150	*ssuC*	Alkanesulfonate transport protein; membrane component
*S. medicae*	Smed_3151	*tauC-*like	Putative taurine transport system permease protein TauC
*S. medicae*	Smed_2065	*atsA*	Arylsulfatase
**Detoxification**			
*S. meliloti*	SMb21552	*aacC_4_*	Aminoglycoside 6'-N-acetyltransferase
*S. meliloti*	SMb20505	*tfxG*	Trifolitoxin immunity protein
*S. meliloti*	SMc02649	*arsC*	Arsenate reductase protein ArsC
*S. meliloti*	SMc02650	*arsH*	Arsenical resistance protein ArsH
*S. medicae*	Smed_0125	*aacA*	Aminoglycoside N(6')-acetyltransferase type 1
*S. medicae*	Smed_2292	*aphE*	Streptomycin 3''-kinase
*S. medicae*	Smed_5053	*arsH*	Arsenate resistance protein ArsH
*S. medicae*	Smed_5054	*arsB*	Arsenite resistance protein ArsB
*S. medicae*	Smed_5055	*arsC*	Arsenate reductase
**C1 metabolism**			
*S. meliloti*	SMa0002	*fdoG*	FdoG formate dehydrogenase-O, alpha subunit
*S. meliloti*	SMa0005	*fdoH*	FdoH formate dehydrogenase-O, beta subunit
*S. meliloti*	SMa0007	*fdoI*	FdoI formate dehydrogenase-O, gamma subunit
*S. meliloti*	SMa0009	*fdhE*	Formate dehydrogenase accessory protein FdhE
*S. meliloti*	SMa0011	*selA*	L-seryl-tRNA(Sec) selenium transferase
*S. meliloti*	SMa0015	*selB*	Selenocysteine-specific elongation factor
*S. meliloti*	SMa0028	*selD*	Selenide, water dikinase
*S. medicae*	Smed_2095	*folD*	Bi-functional; 5,10-methylene-tetrahydrofolate dehydrogenase and cyclohydrolase
*S. medicae*	Smed_2096	*glyA*	Serine hydroxymethyltransferase
**Sugars and polysaccharides**			
*S. meliloti*	SMb20951	*exoI*	Succinoglycan biosynthesis protein ExoI
*S. meliloti*	SMb21416	*ddhA*	Glucose-1-phosphate cytidylyltransferase
*S. meliloti*	SMb21417	*ddhB*	CDP-glucose 4,6-dehydratase
*S. meliloti*	SMb21418		NDP-hexose 3-C-methyltransferase
*S. medicae*	Smed_5910	*otsB*	Trehalose-phosphate phosphatase
**Cellular processes**			
*S. meliloti*	SMc03854	*ftsY*	Putative cell division protein
*S. meliloti*	SMc03044	*motD*	Chemotaxis protein (motility protein D)
*S. medicae*	Smed_1943	*ftsZ*	Cell division protein FtsZ homolog 2
*S. medicae*	Smed_0273	*motD*	Chemotaxis protein motD
**Others**			
*S. meliloti*	SMc04203	*fecI*	Putative RNA polymerase sigma factor FecI protein
*S. meliloti*	SMc04204	*fecR*	Putative IRON transport regulator transmembrane protein
*S. meliloti*	SMc04205		Putative IRON/HEME transport protein
*S. medicae*	Smed_2092	*dsdA*	D-serine dehydratase
*S. medicae*	Smed_3282	*fbpB*	Ferric transport system permease protein FbpB
*S. medicae*	Smed_3284	*fbpC*	Ferric transporter subunit

^a^All genes are presented in Tables S3 and S4 in Additional file 1. ^b^ID of annotated gene in *S. meliloti *1021 or *S. medicae *WSM419.

### Nod factor biosynthetic genes

**Figure 5:**
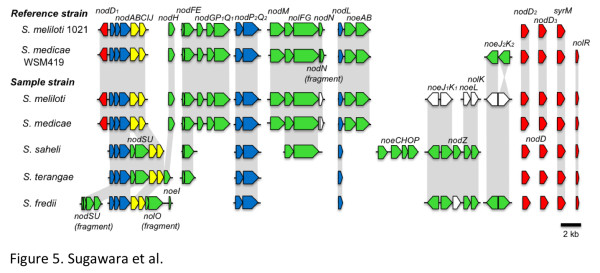
**Gene organization and correlation of Nod factor biosynthetic genes in each *Sinorhizobium *species**. Blue arrows indicate the genes encoding enzymes for Nod factor synthesis commonly detected in all tested *Sinorhizobium *strains. Yellow arrows indicate the genes involved in Nod factor secretion. Green arrows indicate specifically detected genes involved in Nod factor synthesis in an individual species. Red arrows indicate the genes encoding transcriptional regulators of nodulation genes. White arrows indicate genes involved in Nod factor biosynthesis that are not in common.

Most nodulation genes (*nod*, *noe*, and *nol*) are involved in the synthesis of host-specific lipo-chito-oligosaccharide (LCOs) Nod factors that are essential for initiation of the symbiosis [14]. Nearly all rhizobia contain the common nod genes [25], which encode Nod factors secreted from rhizobial cells [14,26]. Figure 5 shows a physical map of Nod factor biosynthesis genes in all five *Sinorhizobium *species. The *S. meliloti *and *S. medicae *strains contain a *nodABCIJ *operon that is closely linked to *nodD_1 _*(encoding positive transcriptional regulator of *nod *genes), whereas *nodD_1 _*of *S. fredii*, *S. saheli and S. terangae *is not closely linked to the common nod genes. *S. meliloti *and *S. medicae *had three copies of *nodD *(*nodD_1_-_3_*) while the other sinorhizobia examined had two copies of *nodD*. Interestingly, the annotated *nodN *(encoding a dehydratase enzyme) was found to be fragmented in many strains of *S. medicae*. The genome of the *S. medicae *WSM419 contained *noeJ_2_K_2_*, whereas *S. meliloti *KH46b had two copies of the *noeJK *genes and a *noeLnolK *gene cluster involved in the fucosylation of the Nod factors at the C-6 position. Since both WSM419 and KH46b strains did not contain a *nodZ *homolog, our data suggest that these strains may not fucosylate their Nod factors. In contrast, *S. saheli *and *S. fredii *strain USDA 207 possessed a complete set of *noeJK-nodZ-noeLK *genes. The *nodZ *in *S. fredii *is also found in *B. japonicum *and is involved in host-specific nodulation of soybean [27].

The sequenced *S. saheli *and *S. terangae *strains contained the *nodSU *genes, which are involved in the *N*-methylation and 6-*O*-carbamoylation of Nod factors [28], inserted between *nodABC *and *nodIJ *genes. In addition, *nolO *and *noeI*, which are involved in 3-*O*-carbamoylation and 2-*O*-methylation of Nod factors, respectively, were localized downstream of the *nodABCIJ *cluster in only the genome of *S. fredii *strains. This organization was similar to that reported for the broad host range *Rhizobium *sp. strain NGR234 [29], but the *nolO *gene was fragmented in the closely related strains USDA 205 and 207. In contrast, the *S. meliloti *and *S. medicae *strains contained *nodGP_1_Q_1_*, *nodM *and *noeAB*, and *S. saheli *had a *noeCHOP *gene cluster, and only *S. fredii *had a *noeI *gene.

Strains of *S. meliloti *are known to synthesize sulfated Nod factors via two copies of *nodPQ *(producing the sulfate donor molecule PAPS) and a *nodH *sulfotransferase. As PAPS is also a central metabolite for sulfate assimilation, *S. meliloti *has additional copies of genes for sulfur metabolism and uses *nodPQ *exclusively for sulfation of Nod factor. In contrast, *S. saheli *and *S. fredii *had only one copy of *nodPQ *and did not contain *nodH*, consistent with the Nod factor structure of *S. saheli *reported earlier [30]. While the *Acacia *symbiont *S. terangae *strain USDA 4894 had a *nodH *gene, it contained fewer Nod factor adornment genes than those seen in other species.

The *nolR *gene, which encodes a negative transcriptional regulator of core Nod factor biosynthesis and is a global regulator in rhizobia [31,32], was detected in all species of *Sinorhizobium*, although the gene in the reference strain *S. meliloti *1021 is not functional [32]. Taken together, these results indicated Nod factor biosynthetic gene content varied among strains of the same species and suggest that LCOs produced by sinorhizobia might be modified in a strain-specific manner. These results are also the first report of genetic organization of nodulation genes in the woody legume symbionts *S. saheli *and *S. terangae*.

### Secretion system gene clusters among *Sinorhizobium *members

**Figure 6:**
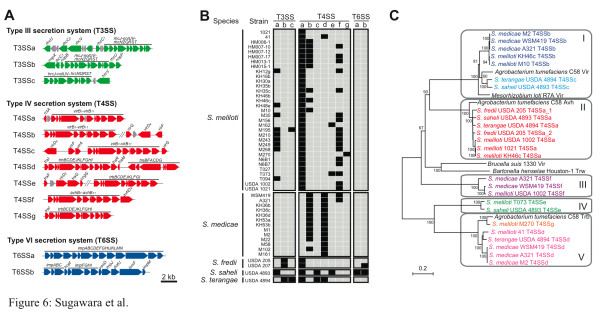
**Gene clusters for type III, IV, and VI secretion systems identified in *Sinorhizobium *species and strains sequenced**. **(a) **Gene organizations of identified type III, IV, and VI secretion system genes. Colored arrows indicate characterized or named genes involved in the protein secretion systems. **(b) **Map showing presence (black plot) or absence (grey plot) of each type of type III, IV, and VI secretion system gene cluster. **(c) **Phylogenetic tree of the *virB *operon from each type IV secretion system gene cluster. Protein sequences of *virB_3_-_5 _*and *virB_8_-_10 _*genes or their orthologs in each type IV secretion system gene cluster were concatenated and used for drawing the tree. Bar indicates number of substitutions per site.

Clusters of genes encoding bacterial type III, IV, and VI protein secretion systems (T3SS, T4SS, and T6SS, respectively) play crucial roles in animal- and plant-bacterial interactions [33]. In rhizobia, these secretion systems are involved in host range determination with their cognate effector proteins modulating host defense reactions [17]. A T3SS gene cluster has been characterized in *Rhizobium *spp. (*S. fredii*) NGR234, *S. fredii *USDA 257 and *S. fredii *HH103 (USDA 207), and T3SS mutants have symbiotic phenotypes [34,35]. However, there are no reports on the roles of T4SS and T6SS systems in sinorhizobial-legume symbioses. Figure 6 shows the structure of the different T3SS, T4SS and T6SS genes found in all the sequenced strains with substantial differences in genomic organization and deduced protein sequences. Notably, the *S. saheli *genome contained T3SS, T4SS, and T6SS gene clusters, as did one of the two *S. fredii *strains, while *S. medicae *strains only contained a T4SS.

Three types of T3SS clusters (types a, b, and c) were identified from several *Sinorhizobium *strains and all clusters contained the canonical *rhcJ-nolUV*-*rhcNQRST *gene cassette (Figure 6a). The T3SSa cluster was detected in nine strains of *S. meliloti *and *S. saheli *USDA 4893 and contained *rhcC_1_*, *rhcC_2_*, *rhcU*, and *rhcV *(Figure 6b). While most of the genes in the main cluster showed 58 to 94% protein identity with the corresponding genes in *Rhizobium *spp. (*S. fredii*) strain NGR234, gene organization of the flanking regions were different. The T3SSb cluster contained the effector genes (*nop*) in *S. fredii *HH103 strain (USDA 207) and was also identified in *S. fredii *USDA 205 and *S. terangae *USDA 4894. Strains having a T3SSc cluster had genes in the main cluster with 40 to 87% protein identity with those of *Rhizobium etli *CIAT 652 and were only observed in the genomes of *S. meliloti *M195 and *S. terangae *USDA 4894. The T3SS types a and c gene clusters found in *S. meliloti*, *S. saheli *and *S. terangae *had a different gene organization from any published *Rhizobium *T3SS clusters and did not contain the well-characterized *nop *genes, encoding T3SS-dependent surface appendage or effector proteins. The unique T3SS apparatus found in these strains may encode novel secretion proteins involved in host-specific interactions.

*Agrobacterium tumefaciens *C58 also uses T4SS for conjugation and DNA transfer [36] and strain C58 possesses three types of T4SS genes: *vir*, *avh*, and *trb*. The *virB *gene of *S. meliloti *1021 (grouped in T4SSa) is involved in conjugation, but is not required for symbiosis with alfalfa [37]. In contrast, *vir *genes of *Mesorhizobium loti *strain R7A are involved in protein translocation and have a host-dependent effect on symbiosis [38]. While seven types of T4SS gene clusters (designated T4SSa-g) were identified in the *Sinorhizobium *genomes (Figure 6a), they were not present in all strains (Figure 6b), suggesting these genes were likely acquired by horizontal gene or plasmid transfer events. To explore the potential function of each *Sinorhizobium *T4SS gene cluster, a phylogenetic tree was created using selected T4SS protein sequences from diverse bacteria known to infect plant and mammalian hosts (Figure 6c). A total of five clades were detected in the phylogenetic tree and T4SSb and T4SSc were present in clade I, including the Vir proteins of *M. loti *R7A and *A. tumefaciens *C58. In contrast, proteins in T4SSa, T4SSd, and T4SSg were in clades II or V and were similar to conjugation transfer proteins Trb or Avh of *A. tumefaciens*. Since the *Sinorhizobium *VirB proteins are similar to the symbiotically effective VirB in *M. loti *R7A, these results indicate that the T4SSb and T4SSc genes in *Sinorhizobium *strains may also influence symbiosis. The T4SSb gene cluster was found in 9 and 11 strains of *S. meliloti *and *S. medicae*, respectively, and the T4SSc cluster was only found in the *Sesbania *and *Acacia *symbionts (*S. saheli *and *S. terangae*), suggesting that the cluster plays a role in host-specific interactions.

The T6SS locus (referred to as *imp*) is a determinant of host specificity in *Rhizobium leguminosarum *[39]. The *S. saheli *strain USDA 4893 had two types of T6SS gene clusters, and T6SSb was also present in *S. fredii *USDA 207. The T6SSa cluster is very similar to that seen in *R*. *leguminosarum *at the amino acid level. No T6SS gene cluster was found in the *S. meliloti*, *S. medicae*, and *S. terangae *strains. Taken together, these results suggest that each sinorhizobial species utilizes different protein secretion strategies to modulate host-specific interactions, although further mutational and functional studies are needed to determine the role of these secretion systems in symbiosis.

### General regulatory systems of T3SS and T4SS genes in rhizobia

In general, the expression of T3SS genes (*rhc and nop*) or T4SS genes (*vir*) is induced by the positive regulators TtsI (for T3SS) and VirA (for T4SS). TtsI and VirA bind to a *tts*- or *vir*-box in the promoter region of T3SS genes (*rhc and nop*) and T4SS genes (*vir*), respectively. In addition, the *ttsI *and *virA *genes have a nod box in front of them, indicating that these genes are likely induced by the NodD protein.

The homologous genes of T3SS effector proteins (NopABCJLMPTX from *S. fredii *NGR234) and the TtsI transcriptional regulator of T3SS genes were searched by BLAST analysis. Results of this analysis indicated that while the *nop *genes and *ttsI *were found in the genome of *S. fredii *USDA 205 and USDA 207 and in *S. terangae *strain USDA4894, which have the T3SSb gene cluster (Table S5 in Additional file 1), they were not found in the genomes of any *S. meliloti *strains. Moreover, a canonical *nod *box consensus sequence was not identified around any region of T3SS-related genes (*rhc*, *nop *and *ttsI*), although tts boxes were found upstream of some *nop *genes in the genomes of *S. fredii *USDA205 and USDA207 and the *S. terangae *strain USDA4894 (Table S6 in Additional file 1), which have the T3SSb cluster.

Blast analyses were used to search the sequenced genomes for genes homologous to those encoding the T4SS effector proteins Msi059 and Msi061 from *M. loti *R7A and a VirA transcriptional regulator of T4SS genes. While the Msi061 homolog was found in the T4SSb and T4SSc gene clusters, Msi059 was not found in the genomes of any of the *Sinorhizobium *strains (Table S7 in Additional file 1). A VirA homolog was only found in the genomes of *S. saheli *strain USDA 4893 and *S. terangae *strain USDA 4894, in the T4SSc cluster (Table 3). In contrast, nod and vir box-like sequences were not identified in the T4SSb and T4SSc clusters of any of the sequenced strains. Taken together, these results suggest that the expression of identified T3SS and T4SS genes might not be regulated by the previously reported nod box inducers. However, further analysis is needed to examine the regulation of these genes.

### Symbiotic phenotypes of T4SSb mutants of *S. meliloti *and *S. medicae *

To further investigate the role of T4SSb in nodulation, deletion mutants of *virB_6 _*to *virB_9_*, predicted to encode essential components of the T4SS apparatus in *S. meliloti *KH46c and *S. medicae *M2, were constructed and inoculated onto nine genotypes of *M. truncatula *and one genotype each of *M. sativa*, *Medicago tricycla *and *Medicago littoralis*. A few symbiotic differences between the wild-type strains and the KH46c and M2 *virB_6_-_9 _*mutants were detected in certain *Medicago *genotypes (Table 2). *M. truncatula *cv. A17 and *M. tricycla *inoculated with the *virB_6_-_9 _*mutant of *S. meliloti *KH46c formed significantly fewer nodules and had lower nodule and plant biomass than that seen in plants inoculated with the wild-type strain. Unexpectedly, however, the *virB_6_-_9 _*mutation in *S. medicae *M2 significantly increased nodule and plant biomass on *M. truncatula *cv. F83005-5. The KH46c *ΔvirB_6_-_9 _*mutant produced about four-fold greater nodule mass on *M. sativa *cv. Agate than did the wild-type strain (Table 2), but had about three- fold less acetylene reduction activity (432 ± 376 μmol C_2_H_4 _produced/h/g nodule dry weight) than the wild-type (1,132 ± 163 μmol C_2_H_4 _produced/h/g nodule dry weight), suggesting a less effective symbiotic interaction. While further experiments are needed to better understand the function of T4SSb in symbiosis, these results indicate that the T4SSb in *Sinorhizobium *may indeed play a role in host specificity. Observations from phenotype tests and gene content differences found in the genome data set suggested that the T4SSb secretion system is likely involved in symbiotic nitrogen fixation with specific *M. truncatula *genotypes. In particular, VirB proteins were postulated as symbiotic effector proteins in *M. loti *R7A [38]. However, we cannot rule out the possibility that other genes are important for host-determination and/or symbiotic efficiency.

**Table 2:** Symbiotic phenotypes of *Medicago *plants inoculated with *virB *mutants of *S. meliloti *KH46c and *S. medicae *M2

Host plant	Inoculated strain	Nodule number^a^	Nodule dry mass (mg)	Plant dry mass (mg)	Plant height (cm)	Chlorophyll content (SPAD unit)
*M. truncatula *	KH46c wild-type	79	6.6	208	12.2	44
A17	KH46c Δ*virB_6_-_9_*	38*	4.3*	145*	9.5*	43
	M2 wild-type	102	8.4	229	11.0	41
	M2 Δ*virB_6_-_9_*	51	6.2*	202	11.2	44
	Uninoculated control	0	0	37	3.3	17
						
*M. truncatula *	KH46c wild-type	35	6.1	174	10.3	42
F83005-5	KH46c Δ*virB_6_-_9_*	24	5.5	158	9.8	39
	M2 wild-type	29	4.9	156	9.5	43
	M2 Δ*virB_6_-_9_*	22	6.7*	243*	10.7*	41
	Uninoculated control	0	0	44	3.3	16
						
*M. tricycla *	KH46c wild-type	24	12.2	315	10.5	36
R108-C3	KH46c Δ*virB_6_-_9_*	12*	9.9	230	10.3	34
	M2 wild-type	11	2.8	33	4.2	19
	M2 Δ*virB_6_-_9_*	12	3.1	33	4.2	21
	Uninoculated control	0	0	26	3.5	16
						
*M. satvia *cv	KH46c wild-type	56	1.6	95	8.5	54
Agate	KH46c Δ*virB_6_-_9_*	42	6.8*	55	7.2	45*
	M2 wild-type	31	2.5	69	13.7	31
	M2 Δ*virB_6_-_9_*	28	2.5	85	14.6	28*
	Uninoculated control	0	0	79	12.5	21

^a^Values are per plant. The asterisk indicates a significant difference compared with the wild-type strain by *t*-test (*P * < 0.05) of three biological replicates.

### Anaerobic denitrification genes

The ability of rhizobia to denitrify depends on the *nap*, *nir*, *nor*, and *nos *gene clusters that encode nitrate-, nitrite-, nitric oxide-, and nitrous oxide-reductases, respectively [40,41]. Denitrification plays an important role in nitrogen-fixing soybean-*Bradyrhizobium japonicum *symbiosis and *S. meliloti *has been shown to denitrify under free-living and symbiotic conditions [41]. Genomic data presented here show that while the genomes of *S. fredii*, *S. saheli*, and *S. terangae *strains contained *napEFDABC*, *nirKV*, and *norECBQD*, they did not have the *nosRZDFYLX *genes that are involved in the terminal step of converting nitrous oxide to N_2_. In contrast, the *nosRZDFYLX *gene cluster was identified in 22 *S. meliloti *strains (Table 3), 19 of which had a complete gene set allowing for the production of N_2 _gas from nitrate.

**Table 3:** Presence of accessory genes involved in polysaccharide biosynthesis, microaerobic denitrification, lithotrophic growth, and organic sulfur utilization in the genomes of each *Sinorhizobium *species

		Gene present in each *Sinorhizobium *species^a^				
		
Gene or gene cluster	Function	*meliloti *(*n *= 33)	*medicae *(*n *= 13)	*fredii *(*n *= 2)	*saheli *(*n *= 1)	*terangae *(*n *= 1)
**Polysaccharide biosynthesis**						
*exoF_2_*	Succinoglycan biosynthesis	7	0	2	0	0
*exoH*	Succinoglycan biosynthesis	33	13	0	0	0
*exoI*	Succinoglycan biosynthesis	33	0	0	1	0
*exoI_2_*	Succinoglycan biosynthesis	11	0	2	0	0
*exoP_2_*	Succinoglycan biosynthesis	7	0	2	0	0
*exoTWV*	Succinoglycan biosynthesis	33	13	0	0	0
*expA_1_-_10-_expGCD_1_D_2_-expE_1_-_8_*	Galactoglucan biosynthesis	33	13	0	0	1
*rkp-*3; *rkpLMNOPQ*	Capsular polysaccharides biosynthesis	4	0	2	0	1
*rkpZ_1_*	Capsular polysaccharides biosynthesis	33	13	1	1	1
*rkpZ_2_*	Capsular polysaccharides biosynthesis	5	0	2	1	1
*rkpT_2_*	Surface polysaccharide export	29	13	1	1	1
*cgmB*	Cyclic β-glucan biosynthesis	1	0	0	0	0
**Microaerobic denitrification**						
*napEFDABC*	Nitrate reductase	32	13	2	1	1
*nirKV*	Nitrite reductase	19	9	2	1	1
*norECBQD*	Nitric oxide reductase	21	9	2	1	1
*nosRZDFYLX*	Nitrous oxide reductase	22	0	0	0	0
**Lithotroph**						
*hupSLCDEFGHJKP-hypABFCDE-hoxX*	Uptake hydrogenase	0	0	0	0	1
*soxYZEF-like*	Sulfur oxidation	7	0	2	0	0
*soxZ*	Sulfur oxidation	33	13	2	0	0
**Organic sulfur utilization^b^**						
I: *ssuDABCE*	Alkanesulfonate degradation	33	13	0	0	1
II: *tauRABCXD*	Taurine degradation	33	13	0	0	0
III: *ssuCBA-atsA-*like	Arylsulfatase	0	13	0	0	0
IV: *tauC-ssuCBA-ats- *like	Arylsulfatase	0	13	0	0	0
V: *ssuADCB*	Alkanesulfonate degradation	0	0	2	0	0

^a^Values in a column indicate number of strains possessing a gene or gene cluster in a species. ^b^The genes in each gene cluster are orthologs of Smed_4212-4216 (I), Smed_4858-4863 (II), Smed_1127-1130 (III), Smed_3146-3151 in *S. medicae *WSM419, and U205v1_247004-247007 (V) in *S. fredii *USDA 205.

### Species differences in organic sulfur utilization genes

The majority of sulfur in agricultural soils is in organic form, such as sulfonates and sulfur-esters [24], and assimilation of these compounds by rhizobia is important for bacterial survival, competition in soils, and during symbiosis [23]. While Koch *et al. *[42] proposed that sulfonate monooxygenase is involved in host-specific adaptation by *B. japonicum*, little is known about organic sulfur utilization in sinorhizobia. Genome annotation indicated the presence of organic sulfur utilization genes (Table 3) and likely species-specific differences in the presence of genes for sulfonate monooxygenases (sulfonate sulfur utilization) or sulfatases (ester-sulfur utilization). *S. meliloti *and *S. medicae *specifically had cluster I (*ssuDABCE *encodes sulfonate transport and desulfonation proteins) and cluster II (*tauRABCXD *encodes taurine uptake and desulfonation proteins). In contrast, only *S. medicae *strains contained clusters III and IV, containing arylsulfatases (ester-sulfur utilization) [43] and *ssuCBA*-like organic sulfur transporter genes (Table 3; Figure S2 in Additional file 2). We tested for sulfatase activity in nodules induced in *Medicago *genotypes (HM011, HM014, HM019, HM028, HM101) by five *S. meliloti *(RM1021, M243, M210, M270, M30) and five *S. medicae *strains (WSM419, M102, M161, A321, M58). With few exceptions, sulfatase activity was greater in nodules induced by *S. medicae *than by *S. meliloti*, averaging 6.1 and 29.4 units/HM011 nodule, respectively. In addition, because *S. medicae *strains commonly have arylsulfatase genes associated with transporter genes (in clusters III and IV), strains of this species may uptake and utilize a wider variety of organosulfur compounds than *S. meliloti*.

### Phenotypic interactions between sequenced *Sinorhizobium *spp. strains and diverse *M. truncatula *genotypes

**Figure 7:**
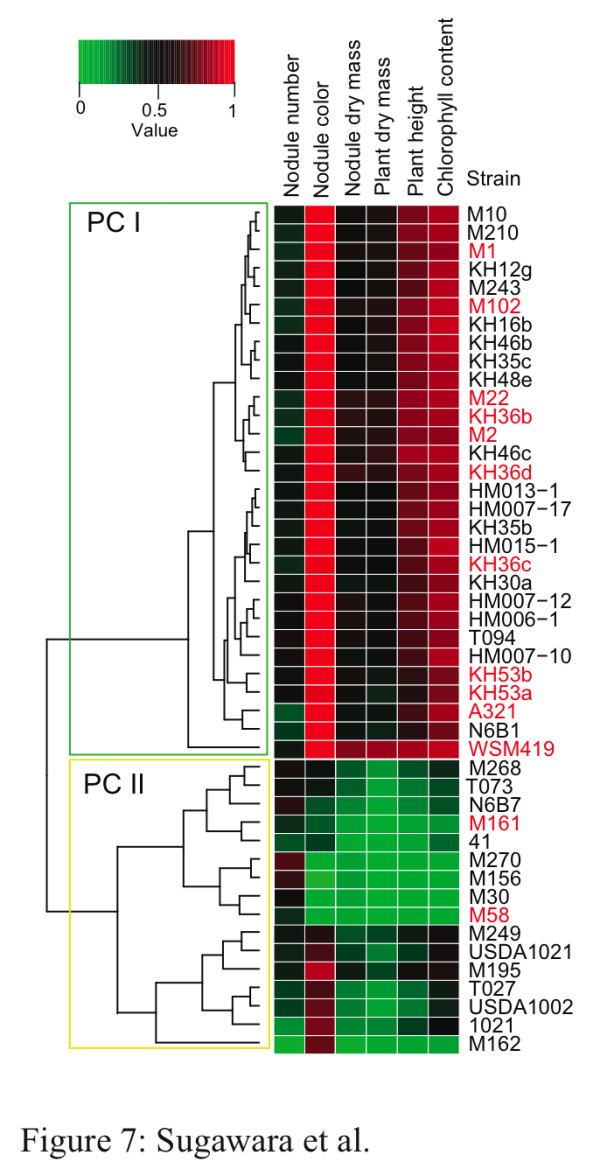
**Symbiotic phenotypes of each *S***. *meliloti *and S. *medicae *strain with *M. truncatula*. Dendrogram and heatmap showing the results of clustering analysis based on the phenotype values. Averaged raw values of each phenotype from three biological replicates were normalized to the range 0 to 1 in each *M. truncatula *genotype. The normalized values were then averaged for 27 genotypes of *M. truncatula*, and clustered. The color in the heatmap indicates the level of value; red indicates the highest and green indicates the lowest value. Black colored names indicate *S. meliloti *strain, and red colored names indicate *S. medicae *strain. PC, phenotype cluster.

We assessed the symbiotic interaction of 46 *S*. *meliloti *or *S*. *medicae *strains with 27 *M*. *truncatula *genotypes. Symbiotic analyses indicated highly significant rhizobial-plant genotype interactions among the tested *Sinorhizobium *strains and *M. truncatula *genotypes (Figure 7; Tables S1 and S8 in Additional file 1). Most strains formed nodules on the roots of all *M. truncatula *genotypes, although *S. meliloti *strain M162 did not form nodules on 17 of 27 *M. truncatula *genotypes. The *noeA *gene, which was characterized as a host-specific nodulation gene [44], was found to be truncated in the nodulation-deficient strain *S. meliloti *M162, suggesting that the failure of this strain to nodulate some *Medicago *genotypes might be caused by a natural mutation in *noeA*. A cluster analysis using normalized and averaged values for each phenotype category obtained from all 27 *M. truncatula *genotypes is presented as a heat map (Figure 7). Strains were divided into phenotype clusters I (PC I) and II (PC II). The PC I included 30 strains that showed high compatibility with *M. truncatula *as measured by the increase in chlorophyll content and plant biomass, significantly more than the 16 strains in the PC II. Strains of both *S. meliloti *and *S. medicae *were present in both PC I and II, suggesting that differences in the symbiotic compatibility with *M. truncatula *were likely caused by strain-specific differences in symbiotic genes.

To investigate the sinorhizobial genes that may affect symbiosis and nitrogen fixation with *M. truncatula*, we searched previously identified symbiosis-related genes in *Sinorhizobium *or other rhizobia from the annotated genome data set of 46 *S*. *meliloti *or *S*. *medicae *strains. The proportion of strains having a full-length gene or gene clusters in each phenotypic cluster were obtained and compared to the proportions in other phenotypic clusters (Table 4). The T4SSb gene cluster (Figure 6) was conserved in 47% of *S. meliloti *and all *S. medicae *strains grouped in PC I; however, it was absent in all strains grouped in PC II (Table 4). In addition, *hemN*, involved in heme biosynthesis, and *nirKV*, *norECBQD*, and *nosRZDFYLX*, involved in microaerobic denitrification, were also conserved in relatively greater numbers of strains grouped in PC I (Table 4). In contrast, the proportion of strain containing previously reported symbiosis-related genes, such as T3SSa, genes involved in polysaccharide biosynthesis, and *acdS *(encoding 1-aminocyclopropane-1-carboxylate deaminase), were not differenct between among PC I and PC II strains. Taken together, these results suggest that protein secretion by the newly identified T4SSb and anaerobic respiration by denitrification might have an important role in symbiotic compatibility with *M. truncatula*.

**Table 4:** Presence of variable length symbiosis-related genes in each phenotype cluster of *S. meliloti *and *S. medicae*

	Species and phenotype cluster (PC)^a^			
	
	*S. meliloti*		*S. medicae*	
		
Gene or gene cluster	I (*n *= 19)	II (*n *= 14)	I (*n *= 11)	II (*n *= 2)
**Nodulation**				
*nodN*	95 (18)	64 (9)	0	0
*noeA*	100 (19)	93 (13)	100 (11)	100 (2)
*noeJ_1_K_1_*	5 (1)	0	0	0
*noeJ_2_K_2_*	0	0	9 (1)	0
*noeLnolK*	5 (1)	0	0	0
**Nitrogen fixation**				
*fixQ*	100 (19)	86 (12)	100 (11)	100 (2)
*fixR*	100 (19)	93 (13)	0	0
*fixU*	95 (18)	79 (11)	100 (11)	100 (2)
*nifD*	100 (19)	100 (14)	100 (11)	50 (1)
*nifE*	100 (19)	100 (14)	90 (10)	100 (2)
**Succinoglycan (EPS I) biosynthesis**				
*exoF_2_*	26 (5)	14 (2)		
*exoI*	95 (18)	100 (14)	0	0
*exoI_2_*	32 (6)	36 (5)	0	0
*exoP_2_*	26 (5)	14 (2)	0	0
*exoW*	100 (19)	93 (13)	100 (11)	100 (2)
**Galactoglucan (EPS II) biosynthesis**				
*expD_2_*	95 (18)	86 (12)	100 (11)	100 (2)
*expE_8_*	95 (18)	100 (14)	100 (11)	100 (2)
**Cyclic β-glucan biosynthesis**				
*cgmB*	0	7 (1)	0	0
**Capusular polysaccharide biosynthesis**				
*rkpLMNOPQ*	16 (3)	7 (1)	0	0
*rkpRSTZ_1_*	100 (19)	93 (13)	100 (11)	100 (2)
*rkpT_2_*	84 (16)	86 (12)	100 (11)	100 (2)
*rkpZ_2_*	16 (3)	14 (2)	0	0
**Type III secretion system**				
T3SSa: *rhc*, *nolUV*	26 (5)	29 (4)	0	0
**Type IV secretion system**				
T4SSa: *rctA*, *vir*	100 (19)	100 (14)	0	0
T4SSb: *vir*	47 (9)	0	100 (11)	0
T4SSd: *tra*, *trb*	0	7 (1)	100 (11)	100 (2)
T4SSe: *tra*, *trb*, *virD_2_*, *cogG *	0	14 (2)	0	0
T4SSf: *avh*	37 (7)	71 (10)	18 (2)	0
T4SSg: *tra*, *trb*	0	7 (1)	0	0
**Denitrification**				
*napEFDABC*	100 (19)	93 (13)	100 (11)	100 (2)
*nirKV*	84 (16)	29 (4)	82 (9)	0
*norECBQD*	84 (16)	29 (4)	82 (9)	0
*nosRZDFYLX*	89 (17)	36 (5)	0	
**Heme biosynthesis**				
*hemA_2_*	16 (3)	29 (4)	0	0
*hemN*	74 (14)	36 (5)	73 (8)	0
**1-Aminocyclopropane-1-carboxylate deaminase**				
*acdS *(Smed_5532 ortholog)	21 (4)	0	36 (4)	100 (2)
*acdS *(Smed_6456 ortholog)	5 (1)	36 (5)	36 (4)	0

^a^The percentage and number (in parentheses) of strains possessing a gene or gene cluster are shown for each species group and phenotype cluster.

## Conclusions

The results of comparative genomics analysis of the *Sinorhizobium *genus provide useful information for understanding the genetic functional features of a wide variety of *Sinorhizobium *species strains, and a tool to better understand incompatibility in legume-rhizobia interactions. The correlation between the presence of T4SS and symbiotic efficiency suggest that each *Sinorhizobium *strain uses a slightly different strategy to obtain maximum compatibility with a host plant. Moreover, these large genomic data sets provide the opportunity to understand the evolution of rhizobia [20] together with mechanisms of host determination, nodulation, and nitrogen fixation. Our overall goal is to combine these data with our previous studies reporting SNPs in *M*. *truncatula *[21] and the sinorhizobia reported here [20] to provide a resource for genome-wide association mapping of genes and traits associated with symbiosis and nodulation. Moreover, the information provided here will be useful to study the population genomics of this bacterium and its evolution with *Medicago*.

## Materials and methods

### Bacteria used in this study

Illumina GAIIx sequencing was used to sequence the genomes of 32 strains of *S. meliloti*, 12 strains of *S. medicae*, 2 strains of *S. fredii*, and 1 strain each of *S. saheli *and *S. terangae *(Table S1 in Additional file 1). The *S. meliloti *and *S*. *medicae *strains were chosen from the USDA-ARS *Rhizobium *Germplasm Collection as representatives of different multi-locus sequence types [45] or obtained from nodules on *M. truncatula *trap hosts inoculated with slurries of soils obtained from several locations in France [46]. Sinorhizobia were also obtained from nodules of seven *M. truncatula *genotypes (HM004, HM006, HM007, HM0013, HM014, HM015 and A17) as trap hosts using Salses soil from France. The type-strains of *S. fredii *(USDA 205), *S. saheli *(USDA 4893) and *S. terangae *(USDA 4894) were chosen from the USDA-ARS *Rhizobium *Germplasm Collection, and *S. fredii *USDA 207 (syn. HH103) was also included. The *Sinorhizobium *strains were grown in TY medium at 30°C. DNA from each strain was used for Illumina library construction and extracted from culture grown cells using the Wizard Genomic DNA Purification kit (Promega Corp. Madison, WI, USA) with further purification by phenol extraction.

### Illumina DNA sequencing

Paired end libraries were generated using Illumina's Phusion-based library kits following the manufacturer's protocols (Illumina, Hayward, CA, USA). Insert sizes averaged 332 nucleotides (range = 245 to 443). Four samples were multiplexed per lane and sequenced on Illumina GAIIx machines and base-called following the manufacturer's protocols. Sequence reads were paired 90-nucleotide reads. Individual samples averaged just over 1 Gb of sequence (range of 724 to 1,584 Mb per genome for *S. meliloti *and *S. medicae *strains) translating into an average and minimum coverage of 174× and 108×, respectively, of the approximately 6.7 Mb genome before aligning reads. Raw reads and derived SNP calls were analyzed previously [20].

Sequences were *de novo *assembled using ABySS [47]. For each strain, several kmers were run and the best resulting assembly was chosen based on assembly contiguity statistics, placement of a subset of high quality read pairs in the assembly with correct spacing, orientation, and comparisons to reference genome sequences.

### Automatic gene annotation and clustering CDSs found in the *Sinorhizobium *genomes

CDSs were predicted using AMIGene (Annotation of Microbial Genomes) software [48] and predicted genes were functionally annotated as described by Vallenet *et al. *[49]. More than 20 bioinformatics methods were used for functional and relational analyses: homology search in a generalist databank (UniProt) and in more specialized databases (COG, InterPro, and PRIAM profiles for enzymatic classification), prediction of protein localization using TMHMM, SignalP and PsortB tools, computation of synteny groups with all available complete and incomplete (WGS section at NCBI) proteomes, and metabolic network reconstruction using Pathway Tools [49]. This fully automated first round of annotation ended with a functional assignment procedure to infer specific function(s) for each individual gene. This functional assignment was first based on annotations of the *S. meliloti *1021 reference genome [50] for strong orthologs (>85% identity over at least 80% of the length of the smallest protein). All data (syntactic and functional annotations and results of comparative analysis) were stored in the relational database SinorhizoScope. Complete sequence data for the 48 *Sinorhizobium *genomes are publicly available via the MaGe interface [51]. The SRA sequences have also been deposited under accession SRA048718 and sequences and annotation data have been deposited in GenBank under project number PRJNA172127.

All protein sequences, including automatic and manually annotated CDSs from the 48 sinorhizobial strains and those of reference strains (*S. meliloti *1021 and *S. medicae *WSM419), were clustered by the CD-HIT algorithm [52] using a 70% cut-off for protein identity. Twenty-eight truncated CDSs in the reference strain genomes and 32 annotated CDSs having less than 11 amino acids identified from all strains were removed from analyses.

### Phylogenetic analyses

*Sinorhizobium *phylogenetic trees were first created based on 645 concatenated protein-coding sequences; genes were included if they were present in a single copy in all strains and the outgroup (*Rhizobium leguminosarum *bv. *trifolii *WSM1325). Homologous sequences were identified in the outgroup by using the MaGe phyloprofile tool to search for bidirectional best hits with at least 70% protein identity across at least 80% of the length of both sequences between the outgroup and *S. meliloti *1021. A phylogenetic tree was also created based on 16S rRNA gene sequences and alignment to reference genomes in GenBank. Distances between strains were calculated using the dnadist program in phylip [53] v3.69 with the F84 model of evolution, and a neighbor-joining tree was assembled using the neighbor program. Support for the splits in the neighbor-joining tree was assessed by constructing neighbor-joining trees on 1,000 bootstrapped datasets created with seqboot, then mapping the support values on to the tree created from the whole dataset using the sumtrees program [54]. The tree was rooted by treating the *R. leguminosarum *strain as an outgroup, and splits with less than 60% support were collapsed to polytomies.

### *Sinorhizobium *symbiotic phenotype assays

The *Sinorhizobium *strains and *Medicago *genotypes used for phenotype analyses are listed in Table S1 in Additional file 1. *Medicago *seeds were prepared as described by Bucciarelli *et al. *[55]. Plant assays were run as a completely randomized block design with three replications in sterile Leonard jar assemblies containing a 1:1 mixture of Sunshine mix #5 (SunGro Horticulture Inc., Vancouver, Canada) and Turface MVP (Profile Product LLC, IL, USA) and inoculated approximately 10^7 ^TY-grown *Sinorhizobium *cells as described previously [56]. Nodulation studies were done at different times, with six plant genotypes tested each time, with one genotype in common. Plants were watered with nitrogen-free plant nutrient solution [55] and incubated in a plant growth chamber at 25°C with a 16-h light condition and at 21°C for 8-h in the dark. Nodule number, color (pink or white), and dry weight, plant dry weight and height, and chlorophyll content of each plant were determined 5 weeks after inoculation. Chlorophyll content in top trifoliate leaves was measured by using a SPAD-502 Chlorophyll Meter (MINOLTA Inc.) and values were averaged. The phenotype data were statistically analyzed by analysis of variance (ANOVA) and Duncan-Waller test using the SAS software package at α = 0.05. A heatmap was created by using default setting of the 'heatmap.2' program in R 2.14.1 software [57].

### Construction of type IV secretion system gene mutants

*S. meliloti *strain KH46c and *S. medicae *strain M2 were selected as recipients for mutation of T4SSb since these strains formed effective nodules on all tested *M*. *truncatula *genotypes. Mobilizable *virB_6_-_9 _*inactivation plasmids were constructed as follows. The 2.9-kb *virB_6_-_9 _*coding regions from both *Sinorhizobium *strains were amplified by PCR using the oligonucleotide primers virB XbaI_F (5'-GCTCTAGAAGTCTGGGCTCGTTTCAGA-3') and virB_XbaI_R (5'-CGTCTAGAGCGGACGTCTTGAGGTAGAA-3') containing the newly created *Xba*I sites (underlined). The PCR products were digested by *Xba*I and followed by ligation into suicide vector pK18mob to create pMS21 (for KH46c *virB*) or pMS22 (for M2 *virB*). These plasmids were digested by *Ssp*I and *Sca*I to delete a 1.6-kb fragment containing the *virB_6 _*to *virB_9 _*coding region, and the Ω cassette from pHP45Ω was inserted to create pMS25 (KH46c *virB*::Ω), or pMS26 (M2 *virB*::Ω). The plasmids pMS25 or pMS26 were introduced into *S. meliloti *KH46c or *S. medicae *M2 by triparental mating. Mutated strains were selected on TY agar plates containing 20 μg of chloramphenicol (Cm) per ml and 100 μg of spectinomycin/streptomycin (Sp/Sm) per ml. Gene replacement, double crossover mutants were verified by their antibiotic resistance phenotype (Cm and Sp/Sm resistant, and neomycin sensitive), and by PCR amplification using primers that spanned the insertion sites.

### Acetylene reduction assay

The nodulated plant roots were removed aseptically with scissors. Detached roots were placed in air-tight 150 ml serum bottles. Three ml of the air volume in each bottle was replaced by pure acetylene gas (99.8%) using hypodermic syringes. The bottles were incubated at room temperature for 60 minutes. The ethylene concentration in each bottle, before and after incubation, was analyzed by gas chromatography using a Nucon-5765 gas chromatograph (AIMIL Instruments, New Delhi, India) equipped with a flame ionization detector (FID) and a Rt-Alumina BOND/Na_2_SO_4 _column (30 m × 0.53 mm) (Restek Corp., Bellefonte, PA, USA). Nitrogen was used as the carrier gas. The operation temperatures for oven, injector, and detector were set at 50°C, 20°C and 104°C, respectively. All the experiments were conducted in triplicate.

### Sulfatase activity test

Enzyme solutions were prepared by crushing 10 nodules aseptically in 150 μl sterilized 0.85% NaCl and the mixture was homogenized by votexing for 15 s. Sulfatase assays were done as previously described [58]. The method was modified by using 50 mM phosphate buffer, pH 7.0, instead of 0.5 M Tris acetate buffer, pH 8.75.

## Abbreviations

CDS: coding sequence; Cm: chloramphenicol; COG: Clusters of Orthologous Groups; LCO: lipo-chito-oligosaccharide; N_2_: dinitrogen; PC: phenotype cluster; Sm: streptomycin; SNP: single nucleotide polymorphism; Sp: spectinomycin; T3SS, T4SS, and T6SS: bacterial type III, IV, and VI protein secretion systems, respectively.

## Competing interests

The authors declare that they have no competing interests.

## Authors' contributions

MS, MJS, NDY, PT and BE wrote the manuscript. MS, BE, LX, JR and RD carried out plant experiments. MS, BE, BB, JM, AKB, ADF, AF, GM and JEW participated in genome sequencing, assembly, and gene annotation. MS, MJS, BE, BB, TU, LX, GP, MJS, CM, DV, AL, ZR, JM, AKB, ADF and BMV carried out analysis of the genome sequences. MJS, NY, PT and BM were the principal investigators (PIs) of this study.

## Supplementary Material

Additional file 1 **Tables S1 to S8**. Click here for file

Additional file 2 **Figure S1 and S2**. Click here for file
