# Supplementary material for: Comparative genomics of the core and accessory genomes of 48 Sinorhizobium strains comprising five genospecies
Source: Genome Biol. 2013 Feb 20;14(2):R17. doi: 10.1186/gb-2013-14-2-r17 (PMC4053727; doi:10.1186/gb-2013-14-2-r17)
Supplement: Additional file 1 — Tables S1 to S8. [file gb-2013-14-2-r17-S1.PDF]

Table S1. *Sinorhizobium* strains used for genome sequencing and plant genotypes used for phenotype studies.

Strain Name	Synonym	Location	Host species
Sinorhizobium meliloti			
USDA 1021		USA	*Medicago sativa*
N6B1	USDA 1978	Nepal	*Medicago falcata*
USDA 1002		USA	*Medicago sativa*
M30		Syria	*Medicago polymorpha*
M270	USDA 1970	Jordan	*Medicago truncatula*
N6B7		Nepal	*Medicago falcata*
M195		Turkey	*Medicago rigidula*
M162		Syria	*Medicago truncatula*
M210		Turkey	*Medicago noeana*
41		Hungary	*Medicago sativa*
M10		Syria	*Medicago blancheana*
T027		Tunisia	*Medicago truncatula*
T073		Tunisia	*Medicago truncatula*
T094		Tunisia	*Medicago truncatula*
M156		Syria	*Medicago rigidula*
M243		Jordan	*Medicago rotata*
M249		Jordan	*Medicago truncatula*
M268		Jordan	*Medicago orbicularis*
KH12g		France	*Medicago truncatula*
KH16b		France	*Medicago truncatula*
KH30a		France	*Medicago truncatula*
KH35b		France	*Medicago truncatula*
KH35c		France	*Medicago truncatula*

Medicago truncatula* plants	
Cultivar name	Hapmap No.
SA22322	HM001
SA28064	HM002
ESP105-L	HM003
DZA045-6	HM004
DZA315-16	HM005
F83005-5	HM006
Salses71B	HM007
DZA012-J	HM008
GRC020-B	HM009
SA24714	HM010
DZA327-7	HM011
SA26063	HM012
Salses42B	HM013
DZA233-4	HM014
F11013-3	HM015
SA09707	HM016
A10	HM017
A20	HM018
Borung	HM019
TN1.11	HM020
TN1.21	HM021
TN6.18	HM023

KH46b		France	*Medicago truncatula*
KH46c		France	*Medicago truncatula*
KH48e		France	*Medicago truncatula*
HM006-1		France	*Medicago truncatula*
HM007-10		France	*Medicago truncatula*
HM007-12		France	*Medicago truncatula*
HM007-17		France	*Medicago truncatula*
HM013-1		France	*Medicago truncatula*
HM015-1		France	*Medicago truncatula*
Sinorhizobium medicae			
M161		Syria	*Medicago noeana*
M2		Syria	*Medicago blancheana*
M22		Syria	*Medicago polymorpha*
M102	USDA 1964	Syria	*Medicago truncatula*
M58	USDA 1960	Jordan	*Medicago rotata*
M1	USDA 1955	Syria	*Medicago orbicularis*
A321	USDA 1037	Aude, France	*Medicago truncatula*
KH36b		France	*Medicago truncatula*
KH36c		France	*Medicago truncatula*
KH36d		France	*Medicago truncatula*
KH53a		France	*Medicago truncatula*
KH53b		France	*Medicago truncatula*
Sinorhizobium fredii			
USDA 205		China	*Glycine max*
USDA 207	HH103	China	*Glycine max*
Sinorhizobium saheli			
USDA 4893	ORS 609	Senegal	*Sesbania cannabina*
Sinorhizobium terangae			

TN8.3	HM024
F11008-C	HM026
F83005-9	HM027
DZA241-2	HM028
A17	HM101

USDA 4894 ORS 1009 Senegal *Acacia laeta*

Table S2. Genome summary of 48 sequenced *Sinorhizobium* strains.

General Srain Info			General Genome Information											Genomic Objects							
**Strain**	**Synonym**	**Species**	**Seq Length**	**GC%**	**Scaffolds**	**Contigs**	**ratu Regio**	**S Length (**	**enic Lengt**	**coding den**	**nomic Obj**	**CDS total**	**CDS w/o art**	**CDS**	**fCDS**	**misc RNA**	**Total rRN**	**16s copies**	**23s copies**	**5s copies**	**tRNA genes**
41		*meliloti*	7414029	62.05	1	190	12.20	841.10	143.20	87.40	7988	7928	7897	7861	36	17	15	5	5	5	59
HM006-1		*meliloti*	7037904	62.11	1	528	10.08	833.12	150.57	87.24	7622	7560	7554	7542	12	18	3	1	1	1	47
HM007-10		*meliloti*	7164778	62.13	1	216	13.95	848.93	146.83	87.27	7632	7576	7537	7517	20	18	18	6	6	6	59
HM007-12		*meliloti*	7268054	62.07	1	156	13.86	840.95	144.64	87.68	7838	7775	7739	7725	14	19	18	6	6	6	62
HM007-17		*meliloti*	7297325	62.18	1	131	15.16	853.57	142.26	87.79	7779	7710	7677	7650	27	21	18	6	6	6	63
HM013-1		*meliloti*	6979942	62.13	1	168	8.30	849.33	146.97	87.18	7450	7384	7353	7325	28	19	18	6	6	6	60
HM015-1		*meliloti*	7225878	61.93	1	202	9.92	845.72	146.89	87.16	7743	7662	7627	7604	23	19	18	6	6	6	79
KH12g		*meliloti*	6854058	62.15	1	166	9.31	861.94	145.77	87.48	7206	7136	7116	7101	18	18	12	4	4	4	57
KH16b		*meliloti*	7011636	62.21	1	242	13.14	841.77	143.93	87.38	7558	7482	7461	7449	12	16	18	6	6	6	63
KH30a		*meliloti*	6898984	62.13	1	151	8.68	863.67	144.41	87.57	7276	7193	7179	7163	16	16	18	6	6	6	63
KH35b		*meliloti*	7111160	62.17	1	150	11.58	862.86	144.23	87.46	7487	7418	7393	7365	28	15	18	6	6	6	61
KH35c		*meliloti*	7163174	62.17	1	265	18.21	851.45	146.15	87.44	7639	7571	7548	7530	18	16	18	6	6	6	57
KH46b		*meliloti*	6880074	62.18	1	171	14.00	851.70	144.11	87.52	7341	7255	7228	7212	16	35	15	5	5	5	63
KH46c		*meliloti*	7084210	62.09	1	251	9.52	832.22	146.17	87.13	7700	7644	7613	7593	20	14	15	5	5	5	58
KH48e		*meliloti*	6874997	62.13	1	205	7.66	860.74	145.05	87.65	7237	7179	7155	7141	14	16	12	4	4	4	54
M10		*meliloti*	6930126	62.12	1	231	11.01	843.07	150.93	86.93	7432	7368	7337	7319	18	15	18	6	6	6	62
M156		*meliloti*	7024239	62.02	1	229	10.84	846.01	148.64	87.11	7571	7480	7457	7429	28	41	15	5	5	5	58
M162		*meliloti*	7201646	61.99	1	234	12.59	828.49	150.99	86.51	7855	7784	7754	7712	42	19	18	6	6	6	64
M195		*meliloti*	7175208	62.07	1	162	13.88	845.78	148.42	87.01	7685	7617	7589	7561	28	17	18	6	6	6	61
M210		*meliloti*	7147799	62.02	1	190	13.70	848.49	147.85	87.03	7639	7574	7541	7514	27	19	18	6	6	6	61
M243		*meliloti*	7132735	62.11	1	154	15.37	852.18	149.13	87.05	7589	7509	7480	7456	24	25	18	6	6	6	66
M249		*meliloti*	7234147	61.90	1	273	12.79	824.88	153.02	86.65	7913	7849	7820	7796	24	19	15	5	5	5	59
M268		*meliloti*	7270759	61.90	1	339	11.34	831.84	151.50	86.67	7902	7833	7808	7775	33	23	15	5	5	5	56
M270	USDA 197(	*meliloti*	7838390	61.75	1	377	14.45	791.47	154.61	86.06	8929	8858	8827	8790	37	22	18	6	6	6	62
M30		*meliloti*	7190560	62.00	1	227	11.14	833.47	153.21	86.69	7801	7734	7712	7684	28	19	13	5	4	4	57
N6B1		*meliloti*	7600408	61.90	1	297	16.20	821.47	147.36	86.94	8351	8277	8252	8222	30	16	18	6	6	6	65
N6B7		*meliloti*	7698488	61.88	1	275	19.25	821.11	147.75	86.81	8455	8384	8358	8327	31	19	18	6	6	6	60
T027		*meliloti*	7043246	62.12	1	187	13.55	861.06	149.65	87.31	7424	7356	7345	7317	28	15	9	3	3	3	55
T073		*meliloti*	7390403	61.98	1	266	16.31	845.96	150.18	87.04	7901	7827	7813	7784	29	17	12	4	4	4	59
T094		*meliloti*	7158954	62.12	1	195	16.51	846.23	146.41	87.20	7705	7634	7612	7578	34	17	15	5	5	5	61
USDA 100	ATCC 993(	*meliloti*	7568286	62.00	1	359	17.54	814.86	149.58	86.80	8335	8268	8261	8229	32	16	6	2	2	2	52
USDA 1021		*meliloti*	7566581	62.07	1	254	18.75	820.12	146.67	86.99	8303	8235	8211	8190	21	15	15	5	5	5	62
A321	USDA 103	*medicae*	7183183	60.98	1	221	10.80	834.32	145.31	87.27	7761	7684	7670	7650	20	17	15	5	5	5	59
KH36b		*medicae*	6932858	61.09	1	178	12.13	834.73	143.70	87.47	7527	7459	7434	7416	18	12	18	6	6	6	63
KH36c		*medicae*	7240526	61.14	1	179	20.06	835.63	141.85	87.53	7864	7787	7764	7742	22	14	18	6	6	6	68
KH36d		*medicae*	6943414	61.10	1	182	12.59	834.77	146.03	87.37	7527	7461	7443	7427	16	13	12	4	4	4	59
KH53a		*medicae*	6889913	61.10	1	175	10.60	829.54	146.26	87.36	7507	7444	7432	7414	18	15	7	3	2	2	53
KH53b		*medicae*	6894703	61.08	1	164	10.58	831.41	143.57	87.46	7526	7460	7393	7417	18	16	15	5	5	5	60
M1	USDA 195	*medicae*	7203177	61.10	1	254	12.21	828.99	150.23	86.79	7802	7720	7704	7678	26	16	18	6	6	6	64
M102	USDA 196	*medicae*	7172130	61.12	1	242	12.49	816.17	145.64	86.84	7932	7852	7835	7803	32	15	18	6	6	6	64
M161		*medicae*	7260501	61.09	1	221	15.16	835.22	138.78	87.64	7917	7815	7802	7770	32	38	15	5	5	5	62
M2		*medicae*	7174808	61.10	1	242	11.67	825.52	144.57	87.23	7887	7807	7789	7765	24	16	18	6	6	6	64
M22		*medicae*	7492331	60.95	1	214	19.65	824.20	146.91	87.01	8230	8143	8128	8106	22	19	18	6	6	6	65
M58	USDA 196(	*medicae*	7019731	61.09	1	162	10.58	823.34	146.13	87.05	7729	7647	7629	7605	24	18	18	6	6	6	64
USDA 205		*fredii*	7177416	62.16	1	255	12.97	839.05	150.78	86.84	7694	7628	7609	7589	20	19	15	5	5	5	51
USDA 207	HH103	*fredii*	6962831	62.22	1	287	13.70	837.58	148.01	86.97	7489	7413	7402	7383	19	17	12	2	5	5	58
USDA 489	ORS 609	*sahalense*	6179454	63.54	1	200	9.38	858.85	149.14	87.26	6503	6436	6423	6405	18	17	9	4	4	1	54
USDA 489	ORS 1009	*terangae*	7095078	61.30	1	139	11.13	832.06	158.74	86.12	7661	7577	7562	7528	34	18	18	6	6	6	63

Table S3. *S. meliloti* specific genes (421 genes) against *S. medicae* .

Gene ID[a]	Gene name	Function
SMa0002	*fdoG*	FdoG formate dehydrogenase-O alpha subunit (fragment)
SMa0002	*fdoG*	FdoG formate dehydrogenase-O alpha subunit (fragment)
SMa0005	*fdoH*	FdoH formate dehydrogenase-O, beta subunit
SMa0007	*fdoI*	FdoI formate dehydrogenase-O,gamma subunit
SMa0009	*fdhE*	Protein fdhE homolog
SMa0011	*selA*	L-seryl-tRNA(Sec) selenium transferase
SMa0013		hypothetical protein
SMa0015	*selB*	Selenocysteine-specific elongation factor
SMa0025		hypothetical protein
SMa0026		hypothetical protein
SMa0028	*selD*	Selenide, water dikinase
SMa0078		LacI family transcriptional regulator
SMa0079		ABC transporter, permease
SMa0081		ABC transporter, permease
SMa0082		ABC transporter, periplasmic solute-binding protein
SMa0083		ABC transporter, ATP-binding protein
SMa0085		dehydrogenase
SMa0087		hypothetical protein
SMa0091		hypothetical protein
SMa0113		Sensor histidine kinase
SMa0114		two-component response regulator
SMa0123		hypothetical protein
SMa0146		hypothetical protein
SMa0168		hypothetical protein
SMa0169		putative methyltransferase
SMa0179		Transcriptional regulator, LysR family
SMa0180		epimerase
SMa0189		hypothetical protein
SMa0190		putative transcriptional regulator
SMa0191		hypothetical protein
SMa0193		hypothetical protein
SMa0209		hypothetical protein
SMa0211		hypothetical protein
SMa0214	*kduI*	5-keto-4-deoxyuronate isomerase
SMa0223		putative transcriptional regulator
SMa0224		putative transmembrane-transport protein
SMa0226		hypothetical protein
SMa0228	*gdhA*	glutamate dehydrogenase
SMa0229		hypothetical protein
SMa0232		hypothetical protein

SMa0279		hypothetical protein
SMa0281		MerR family transcriptional regulator
SMa0572		hypothetical protein
SMa0576		Leu or Leu/Val/Ile transport binding protein
SMa0579		adenylate cyclase, putative
SMa0581	*nrtC*	nitrate transport ATP binding protein
SMa0583	*nrtB*	nitrate ABC transporter permease
SMa0585	*nrtA*	nitrate ABC transporter substrate-binding protein
SMa0896		hypothetical protein
SMa0903		hypothetical protein
SMa0907		hypothetical protein
SMa0909		hypothetical protein
SMa0911		hypothetical protein
SMa0914		hypothetical protein
SMa0929	*traG*	conjugal transfer coupling protein TraG
SMa0934	*traA1*	TraA1 conjugal transfer protein
SMa0939		sensor histidine kinase of two-component system
SMa0981	*ntrR2*	NtrR2 transcription regulator
SMa1045		hypothetical protein
SMa1299		Transcriptional regulator, TetR family
SMa1301		putative transmembrane transport protein
SMa1302	*virB11*	VirB11-like ATPase
SMa1303	*virB10*	VirB10-like transmembrane secretion protein
SMa1306	*virB9*	VirB9 type IV secretion protein
SMa1308	*virB8*	VirB8 type IV secretion protein
SMa1311	*virB6*	VirB6 type IV secretion protein
SMa1313	*virB5*	VirB5 type IV secretion protein
SMa1315	*virB4*	VirB4 type IV secretion protein
SMa1318	*virB3*	VirB3 type IV secretion protein
SMa1319	*virB2*	VirB2 type IV secretion protein
SMa1321	*virB1*	VirB1 type IV secretion protein
SMa1322		hypothetical protein
SMa1323	*rctA*	Transcription regulator
SMa1328		MtbA protein
SMa1355		hypothetical protein
SMa1362		putative inner-membrane permease
SMa1363		ABC transporter, permease
SMa1364		ABC transporter, periplasmic solute-binding protein
SMa1488		putative oxidoreductase
SMa1491		Putative oxidoreductase
SMa1493		LysR family transcriptional regulator
SMa1503		hypothetical protein
SMa1515		hypothetical protein

SMa1547	*pcm*	protein-L-isoaspartate(D-aspartate) O-methyltransferase (PCMT)
SMa1583	*cyaF5*	CyaF5 adenylate cyclase
SMa1585		hypothetical protein
SMa1589		hypothetical protein
SMa1591	*cyaI3*	CyaI3 adenylate/guanylate cyclase
SMa1592		oxidoreductase, fragment
SMa1625		LysR family transcriptional regulator
SMa1626		hypothetical protein
SMa1684		Sensory histidine kinase
SMa1686		Two-component response regulator
SMa1704		HYPOTHETICAL 50.8 KDA PROTEIN IN SYRB 5'REGION (ORF3)
SMa1726		putative transcriptional regulator
SMa1727		alpha/beta hydrolase fold protein
SMa1729	*proX*	ABC transporter, periplasmic solute-binding protein
SMa1731	*betB2*	Betaine aldehyde dehydrogenase 2
SMa1732		hypothetical protein
SMa1734		hypothetical protein
SMa1735		oxidoreductase
SMa1736		LysR family transcriptional regulator
SMa1737		UPF0324 membrane protein RA0957
SMa1740		Siderophore-interacting protein
SMa1741		ABC transporter, ATP-binding protein
SMa1742		ABC transporter, permease, Fe3+-siderophore transport system
SMa1745		ABC transporter, permease, Fe3+-siderophore transport system
SMa1746		putative iron uptake protein
SMa1747		TonB-dependent siderophore receptor
SMa1749		putative transcriptional regulator
SMa1753		Putative Transporter
SMa1754		ABC transporter ATP-binding protein
SMa1755		ABC transporter periplasmic solute-binding protein
SMa1776		hypothetical protein
SMa1789	*cyaI4*	CyaI4 adenylate/guanylate cyclase
SMa1811		hypothetical protein
SMa1817		hypothetical protein
SMa1825		Transcriptional regulator
SMa1828		putative transcriptional activator
SMa1959		hypothetical protein
SMa1960		hypothetical protein
SMa2231		hypothetical protein
SMa2287		putative LysR-family transcriptional regulator
SMa2289		hypothetical protein
SMa2291		dehydrogenase
SMa2297		hypothetical protein

SMa2303		ROK family transcriptional regulator
SMa2313		Oxidoreductase
SMa2323		hypothetical protein
SMa2347		hypothetical protein
SMb20009		transcriptional regulator protein
SMb20010		hypothetical protein
SMb20033		transcriptional regulator
SMb20034		ABC transporter integral membrane protein
SMb20035		ABC transporter membrane protein
SMb20036		ABC transporter substrate-binding protein
SMb20037	*aroE*	shikimate 5-dehydrogenase
SMb20061		hypothetical protein
SMb20063		hypothetical protein
SMb20104		hypothetical protein
SMb20118		hypothetical protein
SMb20121		hypothetical protein
SMb20213		hypothetical protein
SMb20214		oxidoreductase
SMb20215		transcriptional regulator protein
SMb20235		sugar ABC transporter ATP-binding protein
SMb20301		dehydgrogenase
SMb20354		hypothetical protein
SMb20394		hypothetical protein
SMb20395		dehydrogenase
SMb20396		dehydrogenase
SMb20397		oxidoreductase
SMb20402		alcohol dehydrogenase cytochrome c subunit precursor protein
SMb20403		oxidoreductase subunit protein
SMb20404		aldehyde dehydrogenase
SMb20453		gluconolactonase precursor protein
SMb20492		short chain oxidoreductase
SMb20493		short chain dehydrogenase
SMb20494		transcriptional regulator protein
SMb20505	*tfxG*	trifolitoxin immunity protein
SMb20539	*cyaF6*	adenylate cyclase
SMb20556		hypothetical protein
SMb20586		IclR family transcriptional regulator
SMb20587		CoA-transferase subunit A protein
SMb20588		CoA-transferase subunit B protein
SMb20597		hypothetical protein
SMb20628		hypothetical protein
SMb20648		oxidoreductase
SMb20655		hypothetical protein

SMb20690		hypothetical protein
SMb20691		hypothetical protein
SMb20692	*idnO1*	5-keto-D-gluconate 5-reductase
SMb20694		hypothetical protein
SMb20851		SorC family transcriptional regulator
SMb20852		sugar kinase
SMb20853		sugar-alcohol dehydrogenase
SMb20854		sugar uptake ABC transporter permease
SMb20855	*rbsA3*	Ribose import ATP-binding protein RbsA 3
SMb20856		sugar uptake ABC transporter substrate-binding protein precursor
SMb20857	*pgiA2*	Putative glucose-6-phosphate isomerase 2
SMb20868		two-component sensor histidine kinase
SMb20881		hypothetical protein
SMb20883		hypothetical protein
SMb20900		hypothetical protein
SMb20924	*abfA*	alpha-L-arabinofuranosidase
SMb20951	*exoI*	Succinoglycan biosynthesis protein exoI
SMb20966	*lacZ2*	beta-galactosidase
SMb20967		LacI family transcriptional regulator
SMb20968		hypothetical protein
SMb20969		sugar uptake ABC transporter permease
SMb20970		sugar uptake ABC transporter permease
SMb20971		sugar uptake ABC transporter substrate-binding protein precursor
SMb20972		sugar uptake ABC transporter ATP-binding protein
SMb20973		hypothetical protein
SMb20974		oxidoreductase
SMb20975		amino acid uptake ABC transporter permease
SMb20976		amino acid uptake ABC transporter substrate-binding protein precursor
SMb20977		LacI family transcriptional regulator
SMb21025		hypothetical protein
SMb21042		Peptidase T-like protein RB0614
SMb21044		ATP-dependent DNA ligase
SMb21167		reverse transcriptasematurase
SMb21173		hypothetical protein
SMb21178		membrane protein, methyltransferase
SMb21209		two-component sensor histidine kinase
SMb21210		two-component response regulator protein
SMb21211		hypothetical protein
SMb21215	*orf23*	dipeptide uptake ABC transporter substrate-binding protein precursor
SMb21231		glycosyltransferase
SMb21283	*alc*	Probable allantoicase
SMb21289		protein, may interact with ubiquitin
SMb21290		NADPH quinone oxidoreductase

SMb21405		isochorismatase
SMb21416	*ddhA*	glucose-1-phosphate cytidylyltransferase
SMb21417	*ddhB*	CDP-glucose 4,6-dehydratase
SMb21418		NDP-hexose 3-C-methyltransferase
SMb21425		hypothetical protein
SMb21427		acetyltransferase
SMb21439		translation initiation inhibitor protein
SMb21491		hypothetical protein
SMb21508	*dapA3*	dihydrodipicolinate synthetase
SMb21545		two-component response regulator protein
SMb21546		two-component sensor histidine kinase
SMb21552	*aacC4*	aminoglycoside 6'-N-acetyltransferase, amikacin resistance protein
SMb21556		aldehyde or xanthine dehydrogenase
SMb21557		aldehyde or xanthine dehydrogenase, molybdopterin binding subunit protein
SMb21558		aldehyde or xanthine dehydrogenase, iron-sulfur subunit protein
SMb21559		AraC family transcriptional regulator
SMb21560		two component sensor histidine kinase
SMb21561		two component response regulator protein
SMb21562		hypothetical protein
SMb21563		transcriptional regulator protein
SMb21564		hypothetical protein
SMb21567		hypothetical protein
SMb21569		transcriptional regulator protein
SMb21574		sensor histidine kinase domain-containing protein
SMb21580		hypothetical protein
SMb21602		sugar uptake ABC transporter permease
SMb21603		sugar uptake ABC transporter permease
SMb21604		sugar uptake ABC transporter substrate-binding protein precursor
SMb21605		sugar uptake ABC transporter ATP-binding protein
SMc00027		hypothetical protein
SMc00068		hypothetical protein
SMc00099	*phnA*	alkylphosphonate uptake protein
SMc00104		hypothetical protein
SMc00117		hypothetical protein
SMc00158		hypothetical protein
SMc00159		putative signal peptide protein
SMc00163		putative transcription regulator protein
SMc00164		hypothetical protein
SMc00178		hypothetical protein
SMc00190		hypothetical protein
SMc00193		hypothetical protein
SMc00276		hypothetical protein
SMc00277		hypothetical protein

SMc00278		putative transcription regulator protein
SMc00279		hypothetical protein
SMc00340		putative oxidoreductase protein
SMc00379		putative oxidoreductase protein
SMc00389		hypothetical protein
SMc00391		hypothetical protein
SMc00393		hypothetical protein
SMc00399	*corA1*	magnesium/cobalt transporter CorA
SMc00430		putative oxidoreductase protein
SMc00686		hypothetical protein
SMc00687		hypothetical protein
SMc00718		hypothetical protein
SMc00739		hypothetical protein
SMc00740		hypothetical protein
SMc00798		hypothetical protein
SMc00808	*chrA*	chromate transporter
SMc00816		hypothetical protein
SMc00823		hypothetical protein
SMc00854		hypothetical protein
SMc00899		hypothetical protein
SMc00911		hypothetical protein
SMc00953		putative transcription regulator protein
SMc00954		hypothetical protein
SMc00955		hypothetical protein
SMc00978		hypothetical protein
SMc01000		hypothetical protein
SMc01091		hypothetical protein
SMc01176		hypothetical protein
SMc01177		hypothetical protein
SMc01199		hypothetical protein
SMc01216		hypothetical protein
SMc01259		hypothetical protein
SMc01416		hypothetical protein
SMc01434		hypothetical protein
SMc01448		hypothetical protein
SMc01489		putative signal peptide protein
SMc01521	*ntrR1*	nitrogen regulatory protein
SMc01545		hypothetical protein
SMc01581		hypothetical protein
SMc01702		putative oxidoreductase protein
SMc01713		hypothetical protein
SMc01724		hypothetical protein
SMc01749		replicative DNA helicase

SMc01757		hypothetical protein
SMc01774		hypothetical protein
SMc01776		putative auxin-binding protein
SMc01790		putative glycosyltransferase protein
SMc01797		putative acetyltransferase protein
SMc01944		putative haloperoxidase protein
SMc01945		putative transcription regulator protein
SMc01988		hypothetical protein
SMc01991		short chain dehydrogenase
SMc01992		Putative D-xylulose reductase
SMc02022		putative transcription regulator protein
SMc02066	*tatB*	Sec-independent protein translocase protein tatB homolog
SMc02156		hypothetical protein
SMc02161		putative transport transmembrane protein
SMc02394		hypothetical protein
SMc02406		hypothetical protein
SMc02431		hypothetical protein
SMc02439		hypothetical protein
SMc02461		hypothetical protein
SMc02476		hypothetical protein
SMc02477		hypothetical protein
SMc02493		hypothetical protein
SMc02591		hypothetical protein
SMc02595	*metB*	cystathionine gamma-synthase protein
SMc02596		putative transcription regulator protein
SMc02636		hypothetical protein
SMc02647		putative transcription regulator protein
SMc02648		putative transport transmembrane protein
SMc02649	*arsC*	arsenate reductase protein
SMc02650	*arsH*	hypothetical protein
SMc02657		hypothetical protein
SMc02658		hypothetical protein
SMc02715		UPF0110 protein R02377
SMc02736	*tdk*	Thymidine kinase
SMc02865		putative acetyltransferase protein
SMc02890		putative outer membrane receptor protein
SMc02891		putative oxidoreductase transmembrane protein
SMc02987		hypothetical protein
SMc02988		hypothetical protein
SMc03044	*motD*	chemotaxis protein (motility protein D)
SMc03095		hypothetical protein
SMc03136		hypothetical protein
SMc03137		hypothetical protein

SMc03146		putative transport protein
SMc03147		ABC transporter ATP-binding protein
SMc03148		hypothetical protein
SMc03149		hypothetical protein
SMc03166		hypothetical protein
SMc03185		putative NADPH dehydrogenase quinone reductase transmembrane protein
SMc03186		putative transcription regulator protein
SMc03240		putative signal peptide protein
SMc03243		putative alkaline phosphatase transmembrane protein
SMc03244		hypothetical protein
SMc03854	*ftsY*	putative cell division protein
SMc03887		putative amino acid efflux protein
SMc03901		hypothetical protein
SMc04031	*pip2*	putative proline iminopeptidase protein
SMc04032		putative transcription regulator protein
SMc04033	*pip3*	putative proline iminopeptidase protein
SMc04034		peptide ABC transporter permease
SMc04035		peptide ABC transporter permease
SMc04036		ABC transporter ATP-binding protein
SMc04037		peptide ABC transporter
SMc04161		hypothetical protein
SMc04162		putative transcription regulator protein
SMc04172	*cyaH*	adenylate/guanylate cyclase protein
SMc04191		hypothetical protein
SMc04203	*fecI*	putative RNA polymerase sigma factor FECI protein
SMc04204	*fecR*	putative IRON transport regulator transmembrane protein
SMc04205		putative IRON/HEME transport protein
SMc04206		putative hemolysin-type calcium-binding protein
SMc04225		hypothetical protein
SMc04264		putative transcription regulator protein
SMc04291		putative L-sorbosone dehydrogenase (SNDH) protein
SMc04312		hypothetical protein
SMc04326		putative aminotransferase protein
SMc04327		hypothetical protein
SMc04334		putative formyltransferase protein
SMc04350		putative multidrug efflux system transmembrane protein
SMc04351	*macB*	Macrolide export ATP-binding/permease protein MacB
SMc04361		putative hydantoin racemase protein
SMc04362		putative transport protein
SMc04363		hypothetical protein
SMc04404		putative amino acid efflux transmembrane protein
SMc04463		hypothetical protein
RHIME0018		hypothetical protein

RHIME0057	hypothetical protein
RHIME0076	conserved hypothetical protein
RHIME0112	conserved hypothetical protein
RHIME0117	conserved hypothetical protein
RHIME0118	hypothetical protein
RHIME0126	hypothetical protein
RHIME0182	hypothetical protein
RHIME0220	Epoxide hydrolase (EC 3.3.2.3) (fragment)
RHIME0266	conserved hypothetical protein
RHIME0278	Chitin-binding lectin 1 precursor (PL-I) (fragment)
RHIME0492	hypothetical protein
RHIME0583	hypothetical protein
RHIME0630	conserved hypothetical protein
RHIME0669	conserved hypothetical protein
RHIME0756	hypothetical protein
RHIME0913	conserved hypothetical protein
RHIME0942	conserved hypothetical protein
RHIME0970	hypothetical protein
RHIME1066	hypothetical protein
RHIME1077	hypothetical protein
RHIME1133	hypothetical protein
RHIME1163	hypothetical protein
RHIME1206	conserved hypothetical protein; putative exported protein
RHIME1235	putative regulatory protein
RHIME1311	hypothetical protein
RHIME1345	hypothetical protein
RHIME1347	hypothetical protein
RHIME1428	hypothetical protein
RHIME1477	conserved hypothetical protein
RHIME1573	conserved hypothetical protein
RHIME1594	hypothetical protein
RHIME1667	hypothetical protein; putative exported protein
RHIME1698	hypothetical protein
RHIME2111	hypothetical protein
RHIME2128	hypothetical protein
RHIME2314	hypothetical protein
RHIME2431	hypothetical protein

[a] ID of annotated gene in *S. meliloti* 1021.

Table S4. *S. medicae* specific genes (396 genes) against *S. meliloti* .

Gene ID[a]	Gene name	Function
Smed_0019		conserved protein of unknown function
Smed_0086		YadA domain protein
Smed_0087		Invasion associated locus B family protein
Smed_0088		conserved protein of unknown function
Smed_0125	*aacA*	Aminoglycoside N(6')-acetyltransferase type 1
Smed_0131		transposase
Smed_0183		ABC transporter ATP-binding protein
Smed_0184		Transcriptional regulator, GntR family
Smed_0185		Alpha/beta hydrolase fold-3 domain protein
Smed_0186		Extracellular solute-binding protein family 1
Smed_0187		Binding-protein-dependent transport systems inner membrane component
Smed_0188		Binding-protein-dependent transport systems inner membrane component
Smed_0189		Xylose isomerase domain protein TIM barrel
Smed_0190		Oxidoreductase domain protein
Smed_0204		conserved exported protein of unknown function
Smed_0214		conserved protein of unknown function
Smed_0273	*motD*	Chemotaxis protein motD
Smed_0316		conserved protein of unknown function
Smed_0347		conserved protein of unknown function
Smed_0358		conserved membrane protein of unknown function
Smed_0359		Transcriptional regulator, TetR family
Smed_0360		conserved protein of unknown function
Smed_0362		conserved hypothetical protein; putative membrane protein
Smed_0364		GntR domain protein
Smed_0376		transposase
Smed_0380		conserved protein of unknown function
Smed_0419		Porin
Smed_0468		putative plasmid stability protein y4jJ
Smed_0469		putative plasmid stability protein y4jK
Smed_0494		Plasmid stabilization system (fragment)
Smed_0506		Serine-type D-Ala-D-Ala carboxypeptidase
Smed_0508		conserved membrane protein of unknown function
Smed_0583		conserved protein of unknown function
Smed_0597		Homocysteine S-methyltransferase
Smed_0600		Transcriptional regulator, LysR family
Smed_0601		conserved exported protein of unknown function
Smed_0667		conserved protein of unknown function
Smed_0749		Thiamine pyrophosphate protein TPP binding domain protein
Smed_0750		Cobalamin synthesis protein P47K
Smed_0751		Amidohydrolase 3
Smed_0752		conserved protein of unknown function
Smed_0753		Transcriptional regulator, LysR family
Smed_0754		Putative Spermidine/putrescine ABC transporter (ATP binding protein)
Smed_0755		ABC transporter, binding protein
Smed_0756		Binding-protein-dependent transport systems inner membrane component
Smed_0757		Binding-protein-dependent transport systems inner membrane component
Smed_0849		putative D-xylulose reductase
Smed_0910		FAD-dependent pyridine nucleotide-disulphide oxidoreductase
Smed_0917		Regulatory protein LacI
Smed_0964		conserved exported protein of unknown function
Smed_1044		conserved protein of unknown function
Smed_1128		Putative ABC transporter, ATP-binding protein

Smed_1129		conserved exported protein of unknown function
Smed_1130		Sulfatase
Smed_1260		putative oxidoreductase, aldo/keto reductase family
Smed_1261		putative transcriptional regulatory protein, LysR family
Smed_1278		conserved protein of unknown function
Smed_1279		Type III effector Hrp-dependent outers
Smed_1284		conserved protein of unknown function
Smed_1350		protein of unknown function
Smed_1351		conserved protein of unknown function
Smed_1376		conserved protein of unknown function
Smed_1391	*yfmF*	Fe(3+)-citrate import ATP-binding protein yfmF
Smed_1392		Transport system permease protein
Smed_1393		Transport system permease protein
Smed_1394		Periplasmic binding protein
Smed_1395		Transcriptional regulator, LysR family
Smed_1428		Methyltransferase type 12
Smed_1538		Chemotaxis sensory transducer
Smed_1564		Ankyrin
Smed_1586		Sel1 domain protein repeat-containing protein
Smed_1611		Extensin family protein
Smed_1698		LmbE family protein
Smed_1699		putative ABC transporter, ATP-binding protein; putative carbohydrate transporter (maltose malK-like)
Smed_1700		Binding-protein-dependent transport systems inner membrane component
Smed_1701		Binding-protein-dependent transport systems inner membrane component
Smed_1702		Extracellular solute-binding protein family 1
Smed_1704		Mandelate racemase/muconate lactonizing enzyme
Smed_1707		Short-chain dehydrogenase/reductase SDR
Smed_1708		Transcriptional regulator, GntR family
Smed_1742	*fnrN*	putative transcriptional activator
Smed_1757		conserved hypothetical protein; putative membrane protein
Smed_1809		Methyltransferase FkbM family
Smed_1810		conserved protein of unknown function
Smed_1843		conserved protein of unknown function
Smed_1844		conserved exported protein of unknown function
Smed_1845		conserved protein of unknown function
Smed_1853		Chromosomal replication initiator DnaA domain
Smed_1856		conserved protein of unknown function
Smed_1943	*ftsZ*	Cell division protein ftsZ homolog 2
Smed_1956		Methyltransferase type 12
Smed_1975		conserved protein of unknown function
Smed_1978		conserved membrane protein of unknown function
Smed_1986		conserved exported protein of unknown function
Smed_1992		conserved protein of unknown function
Smed_2038		Oxidoreductase domain protein
Smed_2039		Regulatory protein LacI
Smed_2040		conserved protein of unknown function
Smed_2041		ABC transporter binding protein
Smed_2042		Monosaccharide-transporting ATPase
Smed_2043		ABC transporter related
Smed_2062		Binding-protein-dependent transport systems inner membrane component
Smed_2063		Binding-protein-dependent transport systems inner membrane component
Smed_2064		Extracellular solute-binding protein family 1
Smed_2065	*atsA*	Sulfatase
Smed_2066		ABC transporter ATP-binding protein

Smed_2067		Putative transcriptional regulator, RpiR family (fragment)
Smed_2069		Transcriptional regulator, LysR family
Smed_2092	*dsdA*	putative D-serine dehydratase
Smed_2095	*folD*	bifunctional: 5,10-methylene-tetrahydrofolate dehydrogenase; 5,10-methylene-tetrahydrofolate cyclohydrolase
Smed_2096	*glyA*	serine hydroxymethyltransferase
Smed_2097		Transcriptional regulator, LysR family
Smed_2098	*bztA*	Glutamate/glutamine/aspartate/asparagine-binding protein bztA
Smed_2099		Binding-protein-dependent transport systems inner membrane component
Smed_2100		Polar amino acid ABC transporter, inner membrane subunit
Smed_2101		putative amino acid transport protein, ATP-binding protein
Smed_2141		Peptidase M24
Smed_2142	*ugpC*	sn-glycerol 3-phosphate transport protein (ABC superfamily, atp_bind)
Smed_2143		conserved protein of unknown function
Smed_2144		Binding-protein-dependent transport systems inner membrane component
Smed_2145		Binding-protein-dependent transport systems inner membrane component
Smed_2146		Extracellular solute-binding protein family 1
Smed_2147		Periplasmic binding protein/LacI transcriptional regulator
Smed_2148		conserved exported protein of unknown function
Smed_2183		Extracellular solute-binding protein family 5
Smed_2203		protein of unknown function
Smed_2235		conserved protein of unknown function
Smed_2248		Transcriptional regulator, LysR family
Smed_2267		conserved membrane protein of unknown function
Smed_2279		GCN5-related N-acetyltransferase
Smed_2292	*aphE*	Streptomycin 3"-kinase
Smed_2311		ABC transporter ATP-binding protein
Smed_2349		putative MFS family transporter protein
Smed_2376		putative transcriptional regulator, Crp/Fnr family
Smed_2382		putative urease-associated protein
Smed_2391		Peptidase A1 pepsin
Smed_2433		Transcriptional regulator, TetR family
Smed_2434		conserved protein of unknown function
Smed_2542		conserved protein of unknown function
Smed_2597		Beta-lactamase-like protein
Smed_2606		conserved protein of unknown function
Smed_2713		Transcriptional regulator, LysR family
Smed_2720		HicB family protein
Smed_2721		Sel1 domain protein repeat-containing protein
Smed_2725		conserved protein of unknown function
Smed_2748		conserved membrane protein of unknown function
Smed_2790		conserved protein of unknown function
Smed_2793		Peptidase S41
Smed_2838		conserved protein of unknown function
Smed_2862		conserved protein of unknown function
Smed_2877		putative transmembrane protein
Smed_2935		conserved protein of unknown function
Smed_2978		GntR domain protein
Smed_2979		Extracellular solute-binding protein family 3
Smed_2980		Polar amino acid ABC transporter, inner membrane subunit
Smed_2981		putative amino acid transport protein, ATP-binding protein
Smed_2982		Cysteine desulfurase family protein
Smed_2983		D-hydantoinase (Dihydropyrimidinase) (DHPase)
Smed_2987		conserved exported protein of unknown function
Smed_2990		conserved protein of unknown function

Smed_2991		Esterase, PHB depolymerase family
Smed_2999		conserved protein of unknown function
Smed_3004		conserved protein of unknown function
Smed_3091		Signal recognition particle-docking protein FtsY
Smed_3094		putative metallo-hydrolase/oxidoreductase
Smed_3145		Transcriptional regulator, GntR family
Smed_3146		Sulfatase
Smed_3147	*ssuA*	Aliphatic sulfonates family ABC transporter, periplsmic ligand-binding protein
Smed_3148	*ssuB*	Sulfonate ABC transporter, ATP-binding protein
Smed_3149		conserved protein of unknown function
Smed_3150	*ssuC*	Binding-protein-dependent transport systems inner membrane component
Smed_3151		Binding-protein-dependent transport systems inner membrane component
Smed_3152		Pyridoxal 4-dehydrogenase
Smed_3231		Plasmid stabilization system
Smed_3279		Sensor protein
Smed_3280		Two component transcriptional regulator, winged helix family
Smed_3281		conserved exported protein of unknown function
Smed_3282	*fbpB*	Ferric transport system permease protein fbpB
Smed_3284	*fbpC*	ferric transporter subunit ; ATP-binding component of ABC transporter; CP4-6 prophage
Smed_3302		Putative peptide import ATP-binding protein BOV_A0347 (fragment)
Smed_3303	*oppD*	Oligopeptide transport protein (ABC superfamily, ATP-binding protein)
Smed_3304		Binding-protein-dependent transport systems inner membrane component
Smed_3305	*ddpC*	putative D,D-dipeptide transport system permease protein ddpC
Smed_3306		5-oxoprolinase (ATP-hydrolyzing)
Smed_3307		5-oxoprolinase (ATP-hydrolyzing)
Smed_3308		Extracellular solute-binding protein family 5
Smed_3309		Transcriptional regulator, XRE family
Smed_3318		protein of unknown function
Smed_3318		protein of unknown function
Smed_3318		protein of unknown function
Smed_3387		Pyrimidine dimer DNA glycosylase
Smed_3468		Glutathione-dependent formaldehyde-activating GFA
Smed_3511		conserved hypothetical protein; putative Glutathione-dependent formaldehyde-activating enzyme (GFA)
Smed_3513		putative transcriptional regulatory protein, Ars Family
Smed_3520		TspO and MBR like protein
Smed_3523		Polypeptide-transport-associated domain protein ShlB-type
Smed_3524		Filamentous haemagglutinin family outer membrane protein
Smed_3526		Invasion associated locus B family protein
Smed_3548		Variant SH3 domain protein
Smed_3584		RES domain protein
Smed_3612		Adenylyl cyclase class-3/4/guanylyl cyclase
Smed_3613		Alkyl hydroperoxide reductase/ Thiol specific antioxidant/ Mal allergen
Smed_3648		conserved protein of unknown function
Smed_3649	*yqaA*	Inner membrane protein yqaA
Smed_3756		conserved protein of unknown function
Smed_3797		YidE/YbjL duplication domain protein
Smed_3803		conserved exported protein of unknown function
Smed_3829		conserved exported protein of unknown function
Smed_3891		conserved hypothetical protein
Smed_3895		Aminoquinate/shkimate dehydrogenase (fragment)
Smed_3971		DoxX family protein
Smed_3977		Helix-turn-helix-domain containing protein AraC type
Smed_3990		Monooxygenase FAD-binding
Smed_4013		PfkB domain protein

Smed_4014		Cystathionine gamma-synthase
Smed_4015		Binding-protein-dependent transport systems inner membrane component
Smed_4016		Binding-protein-dependent transport systems inner membrane component
Smed_4017		Extracellular solute-binding protein family 1
Smed_4018		Transcriptional regulator, RpiR family
Smed_4034		Alcohol dehydrogenase zinc-binding domain protein
Smed_4050		conserved protein of unknown function
Smed_4053		protein of unknown function
Smed_4068		Major facilitator superfamily MFS_1
Smed_4072		conserved protein of unknown function
Smed_4082		conserved protein of unknown function
Smed_4101		conserved protein of unknown function
Smed_4118		ROK domain containing protein
Smed_4164	*iciA*	Chromosome initiation inhibitor
Smed_4165		conserved membrane protein of unknown function
Smed_4166		Lysine exporter protein (LYSE/YGGA)
Smed_4167		Dihydrodipicolinate synthetase
Smed_4192		ErfK/YbiS/YcfS/YnhG family protein
Smed_4193		conserved protein of unknown function
Smed_4201		Helix-turn-helix-domain containing protein AraC type
Smed_4247		Alanine racemase
Smed_4248		D-isomer specific 2-hydroxyacid dehydrogenase NAD-binding
Smed_4265		conserved protein of unknown function
Smed_4278		putative chemotaxis protein methyltransferase
Smed_4279		Response regulator receiver protein
Smed_4286		Extracellular solute-binding protein family 1
Smed_4287		Binding-protein-dependent transport systems inner membrane component
Smed_4288		Binding-protein-dependent transport systems inner membrane component
Smed_4289	*ugpC*	sn-glycerol-3-phosphate import ATP-binding protein ugpC 1
Smed_4292	*DER*	D-erythrulose reductase
Smed_4293		Periplasmic binding protein/LacI transcriptional regulator
Smed_4294		Cupin 2 conserved barrel domain protein
Smed_4295		conserved protein of unknown function
Smed_4296		Amidase
Smed_4297		Alpha/beta hydrolase fold
Smed_4298		conserved protein of unknown function
Smed_4306		Hemolysin-type calcium-binding region
Smed_4367		conserved exported protein of unknown function
Smed_4413		conserved protein of unknown function
Smed_4415		conserved protein of unknown function
Smed_4428		protein of unknown function
Smed_4437		conserved hypothetical protein; NTF2-like
Smed_4441		conserved protein of unknown function
Smed_4442		Transcriptional regulator, TetR family
Smed_4443		conserved protein of unknown function
Smed_4449		conserved protein of unknown function
Smed_4453		Carboxymuconolactone decarboxylase
Smed_4454		conserved protein of unknown function
Smed_4462		Transcriptional regulator, XRE family
Smed_4463		HipA domain protein
Smed_4567		Glycosyl transferase group 1
Smed_4614		conserved exported protein of unknown function
Smed_4617		Kinase-like protein
Smed_4896		putative adenylate/guanylate cyclase

Smed_4925		conserved membrane protein of unknown function
Smed_4940		conserved membrane protein of unknown function
Smed_5035		conserved protein of unknown function
Smed_5036		Transcriptional regulator, XRE family
Smed_5042		putative Tripeptide aminopeptidase
Smed_5043		conserved protein of unknown function
Smed_5045		conserved protein of unknown function
Smed_5047		conserved protein of unknown function
Smed_5050	*traD*	Conjugal transfer TraD family protein
Smed_5051	*traC*	putative conjugal transfer protein traC
Smed_5053	*arsH*	Arsenate resistance ArsH
Smed_5054	*arsB*	Arsenite resistance protein arsB
Smed_5055	*arsC*	arsenate reductase (Arsenical pump modifier)
Smed_5075		Uncharacterized HTH-type transcriptional regulator y4tD
Smed_5076	*ygeX*	putative diaminopropionate ammonia-lyase
Smed_5084		putative ligase
Smed_5085		Enoyl-CoA hydratase/isomerase
Smed_5086		Methylmalonyl-CoA mutase, large subunit
Smed_5087		conserved protein of unknown function
Smed_5088		Cobalamin B12-binding domain protein
Smed_5089	*dgdA*	2,2-dialkylglycine decarboxylase
Smed_5090	*dgdR*	HTH-type transcriptional regulator dgdR
Smed_5147		Endonuclease/exonuclease/phosphatase
Smed_5150		Activator of Hsp90 ATPase 1 family protein
Smed_5248		Endonuclease/exonuclease/phosphatase
Smed_5257		Glutathione S-transferase domain
Smed_5268		Protein tyrosine phosphatase
Smed_5272	*ycaN*	Uncharacterized HTH-type transcriptional regulator ycaN
Smed_5293		protein of unknown function
Smed_5293		protein of unknown function
Smed_5303		conserved protein of unknown function
Smed_5307		conserved protein of unknown function
Smed_5309		Helix-turn-helix-domain containing protein AraC type
Smed_5310		Oxidoreductase domain protein
Smed_5324		conserved exported protein of unknown function
Smed_5325		Redoxin domain protein
Smed_5351		Sodium/hydrogen exchanger
Smed_5373		Transcriptional regulator, LysR family
Smed_5375	*traI*	putative acyl-homoserine-lactone synthase
Smed_5377	*trbC*	putative conjugal transfer protein trbC
Smed_5387	*traR*	putative transcriptional activator protein traR
Smed_5388	*traM*	putative transcriptional repressor traM
Smed_5389		Uncharacterized HTH-type transcriptional regulator y4dJ
Smed_5391	*traB*	putative conjugal transfer protein traB
Smed_5397		conserved protein of unknown function
Smed_5402		Transcriptional regulator, XRE family
Smed_5410		conserved protein of unknown function
Smed_5420		conserved protein of unknown function
Smed_5427		conserved protein of unknown function
Smed_5672		Diguanylate cyclase/phosphodiesterase
Smed_5683		conserved membrane protein of unknown function
Smed_5689		conserved protein of unknown function
Smed_5690		conserved exported protein of unknown function
Smed_5691		Sterile alpha motif SAM protein

Smed_5692		conserved protein of unknown function
Smed_5693		conserved exported protein of unknown function
Smed_5694		conserved exported protein of unknown function
Smed_5695		Cyclic nucleotide-binding protein
Smed_5697		Transcriptional regulator, TetR family
Smed_5726		Beta-lactamase-like protein
Smed_5804		Transcriptional regulator, XRE family
Smed_5805		conserved protein of unknown function
Smed_5839		conserved protein of unknown function
Smed_5846		Transcriptional regulator, TetR family
Smed_5848		Activator of Hsp90 ATPase 1 family protein
Smed_5878		Major facilitator superfamily MFS_1
Smed_5879		putative transcriptional regulator, AsnC family
Smed_5880		FAD dependent oxidoreductase
Smed_5881		Polar amino acid ABC transporter, inner membrane subunit
Smed_5882		Extracellular solute-binding protein family 3
Smed_5883		Periplasmic binding protein/LacI transcriptional regulator
Smed_5895		Signal transduction histidine kinase
Smed_5898		conserved protein of unknown function
Smed_5910	*otsB*	putative trehalose-phosphate phosphatase
Smed_5915		conserved protein of unknown function
Smed_5939		transposase (fragment)
Smed_6272		transposase (fragment)
Smed_6303		conserved exported protein of unknown function
Smed_6306		conserved protein of unknown function
Smed_6317		conserved exported protein of unknown function
Smed_6318	*alsR*	HTH-type transcriptional regulator alsR
Smed_6319		conserved exported protein of unknown function
SINMW_c0079		protein of unknown function
SINMW_c0413		exported protein of unknown function
SINMW_c0797		protein of unknown function
SINMW_c0868		exported protein of unknown function
SINMW_c0982		protein of unknown function
SINMW_c1084		protein of unknown function
SINMW_c1228		exported protein of unknown function
SINMW_c1296		protein of unknown function
SINMW_c1313		exported protein of unknown function
SINMW_c1327		exported protein of unknown function
SINMW_c1481		protein of unknown function
SINMW_c1779		protein of unknown function
SINMW_c2030		protein of unknown function
SINMW_c2165		Sulfatase
SINMW_c2450		protein of unknown function
SINMW_c2590		protein of unknown function
SINMW_c2607		conserved protein of unknown function
SINMW_c2716		protein of unknown function
SINMW_c2749		protein of unknown function
SINMW_c2916		protein of unknown function
SINMW_c2986		protein of unknown function
SINMW_c3014		exported protein of unknown function
SINMW_c3204		conserved protein of unknown function
SINMW_c3300		protein of unknown function
SINMW_c3541		protein of unknown function
SINMW_c3622		protein of unknown function

SINMW_p10004	exported protein of unknown function
SINMW_p10040	protein of unknown function
SINMW_p10042	protein of unknown function
SINMW_p10226	protein of unknown function
SINMW_p10298	conserved protein of unknown function
SINMW_p10567	PfkB domain protein
SINMW_p10723	protein of unknown function
SINMW_p10898	protein of unknown function
SINMW_p10923	protein of unknown function
SINMW_p11157	protein of unknown function
SINMW_p11523	protein of unknown function
SINMW_p11536	protein of unknown function
SINMW_p20049	protein of unknown function
SINMW_p20233	protein of unknown function
SINMW_p20238	protein of unknown function
SINMW_p20333	protein of unknown function
SINMW_p20407	protein of unknown function
SINMW_p20723	protein of unknown function
SINMW_p20887	protein of unknown function
SINMW_p20894	conserved membrane protein of unknown function

[a] ID of annotated gene in *S. medicae* WSM419.

Supplemental Table S5. The genes encoding T3SS effector proteins or TtsI in the *Sinorhizobium* genome

Strain	Species	Cluster type	nopA	nopB	nopC	nopJ	nopL	nopM	nopP	nopT	nopX	ttsI
KH12g, KH48e, M195, M210, M243, M249, M268, M30, T094 USDA 4893	*S. meliloti*	T3SSa	no hit	no hit	no hit	no hit	no hit	no hit	no hit	no hit	no hit	no hit
USDA205	*S. fredii*	T3SSb	USDA205v1 _190024	USDA205v1 _190011	no hit	no hit	USDA205v1 _190001	USDA205v1 _1020002	USDA205v1 _190022	USDA205v1 _700030	USDA205v1 _190009	USDA205v1 _1320004
USDA207	*S. fredii*	T3SSb	USDA207v1 _2570026	USDA207v1 _2570012	USDA207v1 _2570025	no hit	USDA207v1 _2570001	USDA207v1 _2680002	USDA207v1 _2570023	USDA207v1 _1120008	USDA207v1 _2570010	USDA207v1 _1450008
USDA4894	*S. terangae*	T3SSb, T3SSc	USDA4894v 1_1350038	USDA4894v 1_600021	no hit	no hit	no hit	no hit	USDA4894v1 _600008	USDA4894v1 _600028	USDA4894v 1_600023	USDA4894v 1_600033
M195	*S. melilot*	T3SSc	no hit	no hit	no hit	no hit	no hit	no hit	no hit	no hit	no hit	no hit

The amino acid sequences of NopA, NopB, NopC, NopJ, NopL, NopM, NopP, NopT, NopX and TtsI in *Sinorhizobium fredii* NGR234 were used as queries for BLAST analysis.

Supplemental Table S6. The T3SS related genes located downstream of a *tts*-box.

Strain	Species	Gene located at downstream of tts-box	
		Gene ID	Gene name
USDA205	*S. fredii*	U205v1_190009	*nopX*
		U205v1_190011	*nopB*
		U205v1_190022	*nopP*
		U205v1_190024	*nopA*
USDA207	*S. fredii*	U207v1_2570010	*nopX*
		U207v1_2570012	*nopB*
		U207v1_2570023	*nopP*
		U207v1_2570025	*nopC*
USDA4894	*S. terangae*	U4894v1_600008	*nopP*
		U4894v1_600021	*nopB*
		U4894v1_600023	*nopX*

Supplemental Table S7. Genes encoding T4SS effector proteins or VirA in the *Sinorhizobium* genome.

			msi061 (virF homolog)		virA	
Strain	Species	Cluster type	Gene ID	Identity (%)	Gene ID	Identity (%)
HM006-1	*S. meliloti*	T4SSb	HM006SM1v1_pld4670009	59.0	no hit	-
HM007-10	*S. meliloti*	T4SSb	HM007SM10v1_pld620021	60.2	no hit	-
HM007-12	*S. meliloti*	T4SSb	HM007SM12v1_pld270006	59.0	no hit	-
HM007-17	*S. meliloti*	T4SSb	HM007SM17v1_pld1290052	59.9	no hit	-
HM013-1	*S. meliloti*	T4SSb	HM013SM1v1_psym121004	59.9	no hit	-
HM015-1	*S. meliloti*	T4SSb	HM015SM1v1_pld780012	60.2	no hit	-
KH35c	*S. meliloti*	T4SSb	KH35Cv1_pld1920024	60.2	no hit	-
KH46c	*S. meliloti*	T4SSb	KH46Cv1_pld1830009	59.0	no hit	-
M10	*S. meliloti*	T4SSb	M10v1_pld1910012	60.2	no hit	-
A321	*S. medicae*	T4SSb	A321SMEDv1_840002	59.9	no hit	-
KH36b	*S. medicae*	T4SSb	KH36Bv1_pld1710032	59.9	no hit	-
KH36c	*S. medicae*	T4SSb	KH36Cv1_1580032	59.9	no hit	-
KH36d	*S. medicae*	T4SSb	KH36Dv1_psym1120002	59.9	no hit	-
KH53a	*S. medicae*	T4SSb	KH53Av1_350012	59.9	no hit	-
KH53b	*S. medicae*	T4SSb	KH53Bv1_1020012	59.9	no hit	-
M1	*S. medicae*	T4SSb	M1SMEDv1_850002	59.9	no hit	-
M102	*S. medicae*	T4SSb	M102v1_1800004	60.2	no hit	-
M2	*S. medicae*	T4SSb	M2SMEDv1_260002	59.9	no hit	-
M22	*S. medicae*	T4SSb	M22SMEDv1_1160011	59.9	no hit	-
WSM419	*S. medicae*	T4SSb	Smed_6142	59.9	no hit	-
USDA4893	*S. saheli*	T4SSc	USDA4893v1_600035	42.0	USDA4893v1_600028	48.7
USDA4894	*S. terangae*	T4SSc	USDA4894v1_490022	29.2	USDA4894v1_490023	52.7

Table S8. Nodule number and mass (g) for all combinations of *Sinorhizobium* strain and *Medicago truncatula* genotypes.

Host genotype	HM001		HM002		HM003		HM004	
**Strain**	**Number**	**Mass**	**Number**		**Number**	**Mass**	**Number**	**Mass**
**41**	60.3	0.0032	45.0	0.0043	6.0	0.0000	21.0	0.0011
**A321**	32.7	0.0110	45.0	0.0126	35.7	0.0071	64.3	0.0086
**HM006-1**	44.7	0.0110	63.0	0.0138	24.7	0.0052	98.7	0.0097
**HM007-10**	52.0	0.0089	73.0	0.0149	42.7	0.0062	134.7	0.0094
**HM007-12**	20.3	0.0070	46.7	0.0097	60.7	0.0099	87.7	0.0106
**HM007-17**	37.7	0.0077	55.3	0.0092	29.3	0.0037	87.0	0.0084
**HM013-1**	30.7	0.0077	57.7	0.0085	62.3	0.0092	115.3	0.0108
**HM015-1**	44.3	0.0124	71.7	0.0161	35.3	0.0054	82.7	0.0083
**KH12g**	34.3	0.0091	73.7	0.0158	33.7	0.0054	93.0	0.0078
**KH16b**	28.0	0.0051	68.7	0.0108	42.3	0.0093	63.3	0.0094
**KH30a**	50.7	0.0161	135.7	0.0133	28.3	0.0028	82.0	0.0080
**KH35b**	22.3	0.0059	65.3	0.0094	53.0	0.0102	72.0	0.0094
**KH35c**	28.0	0.0092	44.7	0.0133	36.7	0.0038	71.3	0.0058
**KH36b**	26.7	0.0108	60.7	0.0151	26.0	0.0022	70.7	0.0081
**KH36c**	21.7	0.0076	43.7	0.0082	25.7	0.0053	52.0	0.0106
**KH36d**	25.3	0.0094	50.3	0.0123	57.3	0.0102	95.7	0.0124
**KH46b**	13.0	0.0046	48.0	0.0094	20.7	0.0034	74.7	0.0095
**KH46c**	25.7	0.0082	53.7	0.0113	69.3	0.0103	132.3	0.0141
**KH48e**	23.0	0.0068	51.7	0.0077	16.3	0.0048	54.7	0.0070
**KH53a**	34.3	0.0113	88.7	0.0194	52.7	0.0062	119.3	0.0111
**KH53b**	47.3	0.0168	68.0	0.0133	33.3	0.0051	98.0	0.0145
**M1**	30.3	0.0073	51.0	0.0109	37.3	0.0056	46.7	0.0084
**M10**	29.0	0.0073	45.7	0.0135	55.3	0.0065	76.7	0.0087
**M102**	31.7	0.0145	41.7	0.0260	20.7	0.0044	44.7	0.0066
**M156**	104.7	0.0069	140.7	0.0057	59.0	0.0025	142.3	0.0049
**M161**	48.3	0.0051	85.3	0.0077	38.0	0.0012	110.7	0.0052
**M162**	12.0	0.0010	34.3	0.0026	8.3	0.0004	32.3	0.0034
**M195**	54.3	0.0082	65.7	0.0105	60.3	0.0041	90.7	0.0068
**M2**	19.3	0.0082	42.0	0.0093	39.0	0.0084	74.3	0.0117
**M210**	23.7	0.0084	42.3	0.0097	32.7	0.0048	57.0	0.0095
**M22**	25.7	0.0119	44.7	0.0147	15.0	0.0054	51.3	0.0069
**M243**	32.0	0.0076	66.7	0.0122	35.7	0.0051	48.3	0.0073
**M249**	77.7	0.0018	71.0	0.0144	4.7	0.0000	13.7	0.0005
**M268**	73.3	0.0079	132.0	0.0067	56.0	0.0042	107.7	0.0063

**M270**	95.0	0.0053	123.7	0.0044	38.7	0.0007	162.7	0.0040
**M30**	93.7	0.0059	102.0	0.0062	24.0	0.0006	141.7	0.0047
**M58**	82.0	0.0050	102.7	0.0052	17.0	0.0007	91.0	0.0035
**N6B1**	35.0	0.0079	58.3	0.0139	35.0	0.0030	49.0	0.0059
**N6B7**	92.7	0.0090	156.7	0.0057	41.3	0.0010	127.0	0.0031
**Rm1021**	44.7	0.0098	26.7	0.0034	37.3	0.0028	10.0	0.0000
**T027**	99.3	0.0165	131.0	0.0171	21.3	0.0008	84.3	0.0041
**T073**	67.3	0.0057	158.3	0.0075	59.0	0.0046	152.0	0.0075
**T094**	68.7	0.0170	131.3	0.0261	132.0	0.0109	113.0	0.0104
**USDA 1002**	48.7	0.0106	106.3	0.0102	28.3	0.0013	102.3	0.0076
**USDA 1021**	55.0	0.0121	177.7	0.0241	20.7	0.0052	103.7	0.0075
**WSM419**	20.7	0.0164	121.7	0.0401	40.7	0.0083	126.7	0.0160

The values are means of three biological replicate experiments. Measurements are per plant.

HM005		HM006		HM007		HM008		HM009	
**Number**	**Mass**	**Number**	**Mass**	**Number**	**Mass**	**Number**	**Mass**	**Number**	**Mass**
43.7	0.0038	56.0	0.0004	13.0	0.0004	25.3	0.0026	12.0	0.0018
43.3	0.0083	28.0	0.0082	23.3	0.0039	37.3	0.0162	13.7	0.0133
61.7	0.0135	43.7	0.0127	34.3	0.0046	91.7	0.0210	39.3	0.0166
47.0	0.0130	66.3	0.0261	35.0	0.0039	114.7	0.0219	29.0	0.0123
40.3	0.0106	53.0	0.0195	37.0	0.0043	80.3	0.0156	62.3	0.0144
20.0	0.0057	69.3	0.0177	33.3	0.0038	74.3	0.0191	28.7	0.0150
40.3	0.0105	62.0	0.0072	20.3	0.0037	66.3	0.0208	39.0	0.0090
49.7	0.0142	71.7	0.0266	31.7	0.0048	81.3	0.0178	27.3	0.0133
17.7	0.0152	31.3	0.0162	34.0	0.0043	42.0	0.0154	35.3	0.0153
24.3	0.0096	15.3	0.0066	28.3	0.0025	47.3	0.0156	35.0	0.0114
32.0	0.0077	24.7	0.0077	36.3	0.0033	54.3	0.0165	43.0	0.0078
39.0	0.0111	16.7	0.0044	30.7	0.0040	45.3	0.0134	45.7	0.0098
47.7	0.0142	61.0	0.0179	58.7	0.0070	74.0	0.0181	39.3	0.0104
16.7	0.0085	33.0	0.0082	26.7	0.0065	53.0	0.0237	20.3	0.0204
56.3	0.0128	39.3	0.0112	42.0	0.0058	67.0	0.0333	45.0	0.0144
29.0	0.0152	75.7	0.0228	52.0	0.0065	59.0	0.0273	33.3	0.0161
35.7	0.0105	64.3	0.0179	52.3	0.0039	63.3	0.0221	26.7	0.0087
23.3	0.0092	25.0	0.0071	52.7	0.0052	45.7	0.0135	22.3	0.0124
25.7	0.0096	33.7	0.0069	45.3	0.0041	80.7	0.0228	41.3	0.0134
41.0	0.0096	84.0	0.0136	61.3	0.0057	74.3	0.0228	29.3	0.0105
64.7	0.0203	50.7	0.0081	74.7	0.0054	60.7	0.0209	26.3	0.0149
27.3	0.0138	38.7	0.0154	28.0	0.0066	45.7	0.0136	51.3	0.0162
34.7	0.0115	37.3	0.0115	31.0	0.0052	74.7	0.0205	38.3	0.0098
16.3	0.0127	33.3	0.0143	32.3	0.0045	39.7	0.0244	31.3	0.0114
101.7	0.0029	34.0	0.0004	52.0	0.0016	82.7	0.0037	36.3	0.0033
41.7	0.0040	40.7	0.0045	26.0	0.0017	46.0	0.0034	29.7	0.0022
2.0	0.0001	3.7	0.0006	0.0	0.0000	10.7	0.0008	4.3	0.0005
47.7	0.0095	57.7	0.0111	34.7	0.0047	59.7	0.0115	21.7	0.0089
33.3	0.0091	24.3	0.0078	51.0	0.0078	30.3	0.0212	29.0	0.0155
18.0	0.0076	18.3	0.0040	34.7	0.0065	69.0	0.0134	38.3	0.0160
28.0	0.0101	32.7	0.0141	40.7	0.0058	34.7	0.0099	17.7	0.0201
38.3	0.0101	48.7	0.0202	40.3	0.0045	58.7	0.0121	36.3	0.0141
44.0	0.0021	34.7	0.0000	25.0	0.0001	58.7	0.0125	24.7	0.0080
108.7	0.0059	33.7	0.0002	60.3	0.0027	48.7	0.0092	30.0	0.0024

114.7	0.0028	53.3	0.0000	69.0	0.0025	112.7	0.0047	41.7	0.0028
94.0	0.0030	51.7	0.0033	55.7	0.0029	54.7	0.0030	29.0	0.0026
53.0	0.0015	27.3	0.0016	27.7	0.0014	68.0	0.0038	29.3	0.0022
76.7	0.0129	17.3	0.0004	52.0	0.0045	38.7	0.0145	39.3	0.0077
107.7	0.0030	40.0	0.0008	47.7	0.0026	50.3	0.0051	44.7	0.0033
17.0	0.0062	23.3	0.0068	7.0	0.0016	21.0	0.0039	8.7	0.0060
15.3	0.0051	36.7	0.0029	26.7	0.0026	69.7	0.0039	11.0	0.0026
70.7	0.0032	25.0	0.0003	32.0	0.0015	61.3	0.0033	37.3	0.0025
50.0	0.0142	26.0	0.0013	52.3	0.0064	62.7	0.0145	25.0	0.0059
60.0	0.0036	26.7	0.0000	29.0	0.0017	72.3	0.0110	25.7	0.0041
67.3	0.0194	43.3	0.0118	31.7	0.0025	56.7	0.0056	22.0	0.0080
68.7	0.0407	52.7	0.0319	32.7	0.0051	35.3	0.0157	22.3	0.0269

HM010		HM011		HM012		HM013		HM014	
Number	Mass	Number	Mass	Number	Mass	Number	Mass	Number	Mass
14.3	0.0011	17.3	0.0014	28.7	0.0021	20.7	0.0004	9.7	0.0003
18.7	0.0128	63.7	0.0164	21.3	0.0171	11.7	0.0022	23.3	0.0148
69.0	0.0124	24.3	0.0221	86.7	0.0259	25.3	0.0046	89.7	0.0175
32.7	0.0081	58.0	0.0195	75.0	0.0284	31.3	0.0037	66.7	0.0129
35.0	0.0055	67.7	0.0191	58.0	0.0179	18.0	0.0039	102.0	0.0239
40.3	0.0106	54.7	0.0217	80.3	0.0254	19.3	0.0034	82.0	0.0135
29.7	0.0123	27.3	0.0133	70.3	0.0226	20.3	0.0043	78.3	0.0157
38.7	0.0085	50.7	0.0194	44.7	0.0133	18.3	0.0038	87.3	0.0182
28.7	0.0093	75.3	0.0234	83.0	0.0213	15.3	0.0038	53.0	0.0142
27.0	0.0066	56.7	0.0174	113.0	0.0309	20.0	0.0042	37.0	0.0118
37.7	0.0073	48.3	0.0185	82.3	0.0134	22.7	0.0033	66.3	0.0093
22.7	0.0064	35.0	0.0197	34.7	0.0157	21.0	0.0047	62.3	0.0113
67.0	0.0112	48.0	0.0135	117.7	0.0249	18.7	0.0043	53.3	0.0112
49.7	0.0225	60.7	0.0189	77.0	0.0334	18.7	0.0061	42.3	0.0144
68.5	0.0113	26.7	0.0114	50.7	0.0207	25.0	0.0041	31.0	0.0166
24.0	0.0117	56.7	0.0187	42.3	0.0199	23.3	0.0051	60.7	0.0132
31.0	0.0073	49.7	0.0172	49.7	0.0176	19.0	0.0037	56.7	0.0142
19.7	0.0102	37.0	0.0152	75.7	0.0219	40.0	0.0089	50.0	0.0135
58.3	0.0104	31.0	0.0117	64.3	0.0230	16.3	0.0039	72.0	0.0135
47.7	0.0118	32.0	0.0222	63.7	0.0229	33.0	0.0029	47.0	0.0088
28.3	0.0139	27.3	0.0187	38.7	0.0143	25.3	0.0052	76.0	0.0138
20.7	0.0107	41.3	0.0359	53.7	0.0177	22.0	0.0044	41.3	0.0203
54.7	0.0117	66.0	0.0183	73.0	0.0224	23.7	0.0048	44.3	0.0130
49.3	0.0136	40.0	0.0173	61.0	0.0246	22.3	0.0045	61.7	0.0237
15.3	0.0018	45.7	0.0034	50.3	0.0027	58.7	0.0031	47.0	0.0019
13.7	0.0015	29.0	0.0025	42.0	0.0043	35.3	0.0022	32.3	0.0026
0.0	0.0000	0.0	0.0000	0.0	0.0000	5.0	0.0001	0.0	0.0000
27.0	0.0083	24.3	0.0115	24.0	0.0073	27.0	0.0062	45.0	0.0103
22.7	0.0209	26.0	0.0320	37.7	0.0196	12.0	0.0044	29.0	0.0168
55.3	0.0146	30.0	0.0166	92.0	0.0287	19.7	0.0046	56.3	0.0169
29.3	0.0193	24.0	0.0197	75.3	0.0286	15.7	0.0034	42.0	0.0246
62.0	0.0095	23.7	0.0131	47.3	0.0149	26.7	0.0034	56.3	0.0115
7.3	0.0000	43.3	0.0267	94.3	0.0277	42.7	0.0040	38.7	0.0128
65.0	0.0076	14.7	0.0028	31.3	0.0037	30.3	0.0032	45.7	0.0063

48.0	0.0023	14.0	0.0011	43.0	0.0029	70.0	0.0033	52.3	0.0026
10.3	0.0009	21.7	0.0028	37.3	0.0033	36.0	0.0021	33.3	0.0021
13.7	0.0016	19.0	0.0019	29.0	0.0020	25.3	0.0022	20.0	0.0013
32.0	0.0126	24.0	0.0288	25.7	0.0156	19.7	0.0046	44.0	0.0141
31.3	0.0069	48.3	0.0039	53.3	0.0023	34.0	0.0018	60.7	0.0029
7.0	0.0054	5.7	0.0021	28.7	0.0120	13.3	0.0035	0.7	0.0001
13.7	0.0019	8.7	0.0030	21.0	0.0071	28.0	0.0031	26.7	0.0052
53.7	0.0033	18.3	0.0066	84.7	0.0256	33.3	0.0045	58.7	0.0053
37.7	0.0085	47.3	0.0229	79.0	0.0241	18.0	0.0038	111.3	0.0111
19.3	0.0044	12.7	0.0035	40.7	0.0031	14.3	0.0018	56.3	0.0069
24.7	0.0038	16.3	0.0037	31.7	0.0026	21.0	0.0021	42.0	0.0167
19.3	0.0218	61.3	0.0390	81.0	0.0273	15.3	0.0046	35.3	0.0260

HM015		HM016		HM017		HM018		HM019	
Number	Mass	Number	Mass	Number	Mass	Number	Mass	Number	Mass
4.7	0.0002	12.0	0.0030	22.3	0.0023	11.3	0.0010	39.0	0.0041
14.0	0.0047	10.0	0.0137	9.0	0.0049	18.7	0.0129	20.3	0.0127
15.7	0.0060	32.7	0.0216	3.7	0.0048	20.0	0.0117	34.0	0.0176
22.3	0.0040	50.0	0.0135	10.7	0.0028	30.7	0.0066	33.7	0.0111
24.7	0.0072	52.7	0.0378	5.3	0.0063	23.7	0.0173	42.3	0.0170
19.3	0.0045	54.3	0.0225	8.3	0.0070	21.3	0.0122	18.7	0.0085
18.3	0.0064	42.0	0.0135	7.3	0.0029	35.0	0.0101	21.7	0.0095
14.0	0.0052	41.3	0.0204	5.7	0.0064	22.3	0.0118	28.7	0.0106
25.0	0.0056	41.3	0.0159	15.0	0.0045	41.0	0.0104	34.3	0.0094
23.0	0.0069	28.7	0.0126	3.0	0.0077	14.0	0.0134	36.7	0.0199
26.0	0.0067	60.3	0.0185	5.0	0.0076	24.7	0.0146	31.0	0.0079
14.3	0.0060	37.0	0.0139	9.0	0.0037	11.3	0.0100	26.3	0.0060
24.7	0.0061	36.3	0.0117	1.3	0.0081	19.7	0.0134	32.7	0.0107
13.7	0.0085	43.3	0.0261	9.0	0.0058	23.0	0.0157	32.7	0.0149
18.0	0.0070	53.3	0.0336	5.0	0.0079	17.0	0.0147	20.3	0.0084
25.0	0.0070	35.7	0.0286	6.0	0.0093	27.7	0.0182	37.7	0.0145
28.3	0.0089	75.3	0.0240	10.3	0.0065	18.3	0.0158	44.0	0.0161
23.0	0.0065	40.3	0.0181	3.0	0.0049	22.0	0.0143	39.0	0.0111
27.0	0.0083	35.3	0.0165	13.7	0.0047	47.0	0.0108	37.3	0.0083
26.3	0.0097	49.0	0.0138	22.3	0.0049	48.0	0.0152	31.3	0.0061
30.3	0.0076	40.3	0.0106	11.7	0.0055	18.3	0.0113	48.7	0.0120
10.7	0.0072	23.7	0.0179	14.7	0.0026	21.3	0.0153	19.3	0.0141
18.3	0.0086	24.3	0.0158	5.7	0.0074	26.0	0.0144	45.0	0.0130
14.3	0.0085	38.7	0.0194	14.3	0.0033	35.7	0.0191	23.7	0.0173
40.7	0.0022	32.7	0.0032	40.7	0.0033	64.0	0.0047	60.7	0.0038
18.7	0.0016	30.7	0.0039	7.0	0.0011	15.3	0.0022	27.7	0.0036
0.0	0.0000	0.0	0.0000	0.0	0.0000	0.0	0.0000	0.0	0.0000
30.0	0.0067	52.0	0.0141	4.7	0.0043	23.7	0.0082	35.0	0.0099
18.0	0.0077	29.0	0.0207	14.7	0.0030	18.0	0.0183	19.7	0.0128
17.0	0.0079	53.7	0.0131	3.3	0.0061	24.7	0.0139	41.7	0.0130
13.0	0.0095	51.0	0.0249	10.0	0.0071	31.3	0.0177	27.7	0.0138
15.7	0.0078	58.0	0.0156	9.3	0.0126	20.3	0.0106	57.0	0.0185
47.7	0.0088	53.7	0.0188	14.0	0.0005	27.0	0.0129	81.0	0.0154
21.7	0.0053	35.3	0.0054	1.7	0.0093	44.3	0.0045	99.0	0.0056

49.7	0.0030	57.3	0.0040	16.3	0.0018	51.0	0.0032	73.0	0.0047
45.7	0.0038	63.0	0.0048	27.3	0.0023	47.0	0.0028	31.3	0.0040
17.0	0.0016	34.7	0.0034	11.7	0.0028	23.3	0.0018	38.3	0.0032
26.0	0.0049	36.0	0.0133	7.0	0.0150	17.7	0.0111	20.0	0.0066
32.0	0.0030	72.3	0.0064	10.3	0.0062	40.3	0.0029	54.7	0.0042
5.7	0.0037	20.0	0.0097	1.7	0.0012	14.3	0.0075	10.7	0.0022
15.3	0.0022	10.3	0.0065	8.3	0.0021	25.0	0.0039	20.7	0.0041
16.3	0.0028	28.7	0.0042	10.0	0.0043	33.3	0.0034	51.7	0.0040
22.7	0.0061	103.7	0.0224	10.3	0.0103	20.7	0.0099	41.7	0.0126
13.7	0.0056	49.0	0.0083	12.0	0.0042	13.0	0.0064	36.7	0.0049
15.7	0.0046	59.7	0.0193	12.7	0.0040	12.3	0.0067	40.0	0.0061
18.0	0.0064	50.7	0.0337	10.3	0.0044	22.3	0.0201	17.7	0.0139

HM020		HM021		HM023		HM024		HM026	
Number	Mass	Number	Mass	Number	Mass	Number	Mass	Number	Mass
16.0	0.0011	51.0	0.0028	6.3	0.0002	21.0	0.0005	39.3	0.0023
17.7	0.0086	23.7	0.0247	5.0	0.0007	11.0	0.0050	9.0	0.0083
29.3	0.0287	84.3	0.0388	4.0	0.0013	22.7	0.0085	14.3	0.0077
19.0	0.0071	68.0	0.0204	6.7	0.0011	26.0	0.0089	29.3	0.0087
39.7	0.0248	82.3	0.0315	6.3	0.0015	27.7	0.0078	24.3	0.0088
18.0	0.0111	91.3	0.0313	2.0	0.0012	14.7	0.0054	44.3	0.0068
22.3	0.0100	65.0	0.0243	5.3	0.0010	28.7	0.0081	24.3	0.0119
19.0	0.0086	41.7	0.0199	4.7	0.0016	12.7	0.0074	29.3	0.0097
12.7	0.0139	43.3	0.0230	2.3	0.0015	11.0	0.0064	24.0	0.0075
26.7	0.0094	40.3	0.0240	3.3	0.0012	11.3	0.0065	14.7	0.0085
16.7	0.0129	56.0	0.0128	3.0	0.0018	16.0	0.0070	14.0	0.0061
28.3	0.0057	28.0	0.0248	7.7	0.0012	11.7	0.0124	30.7	0.0080
42.3	0.0159	44.7	0.0141	4.7	0.0013	9.7	0.0060	17.3	0.0083
25.3	0.0155	52.7	0.0189	3.3	0.0015	13.0	0.0149	15.0	0.0114
20.7	0.0104	59.3	0.0289	2.7	0.0003	15.7	0.0054	17.7	0.0077
22.3	0.0100	49.7	0.0317	7.3	0.0010	17.0	0.0174	15.7	0.0165
25.3	0.0119	82.3	0.0261	5.3	0.0014	25.0	0.0081	24.7	0.0101
35.3	0.0134	47.0	0.0162	3.3	0.0010	16.0	0.0095	30.0	0.0164
42.3	0.0188	57.3	0.0162	5.3	0.0012	17.7	0.0122	29.7	0.0111
10.3	0.0060	51.3	0.0273	5.3	0.0001	28.7	0.0098	18.3	0.0114
42.0	0.0155	41.3	0.0174	4.3	0.0006	30.3	0.0091	22.7	0.0122
9.7	0.0117	30.0	0.0287	10.0	0.0008	18.3	0.0101	28.0	0.0111
12.0	0.0138	44.0	0.0193	2.7	0.0013	13.0	0.0065	41.0	0.0106
12.3	0.0132	35.3	0.0285	2.7	0.0002	13.0	0.0100	28.0	0.0060
50.7	0.0031	115.3	0.0071	3.7	0.0002	38.7	0.0023	25.0	0.0035
12.3	0.0033	38.0	0.0080	1.7	0.0002	60.0	0.0044	40.0	0.0036
0.0	0.0000	4.7	0.0006	0.0	0.0000	0.0	0.0000	0.0	0.0000
26.3	0.0125	80.0	0.0181	4.3	0.0004	26.0	0.0130	25.3	0.0071
20.0	0.0149	30.3	0.0203	6.7	0.0005	15.3	0.0160	9.7	0.0096
24.3	0.0134	25.7	0.0248	5.0	0.0019	12.0	0.0061	19.0	0.0071
21.0	0.0204	64.7	0.0306	9.0	0.0011	27.0	0.0084	22.0	0.0123
14.7	0.0120	43.0	0.0211	3.3	0.0015	21.0	0.0076	22.0	0.0067
46.7	0.0108	73.7	0.0002	2.3	0.0006	5.7	0.0001	44.3	0.0059
60.7	0.0047	67.7	0.0078	5.7	0.0016	34.7	0.0027	19.0	0.0047

45.3	0.0028	77.7	0.0007	3.7	0.0002	67.7	0.0040	47.7	0.0026
23.3	0.0027	77.3	0.0074	3.7	0.0002	51.0	0.0039	36.3	0.0046
13.3	0.0022	56.0	0.0045	2.3	0.0002	35.0	0.0022	41.7	0.0032
12.3	0.0108	61.7	0.0234	5.3	0.0005	30.7	0.0107	20.3	0.0072
26.3	0.0027	73.3	0.0003	6.3	0.0014	72.3	0.0033	49.3	0.0040
16.3	0.0004	9.0	0.0102	1.7	0.0004	9.3	0.0024	31.0	0.0029
17.3	0.0062	34.7	0.0110	4.0	0.0001	25.0	0.0036	25.7	0.0032
33.7	0.0087	138.7	0.0115	6.7	0.0011	30.7	0.0026	19.0	0.0042
24.3	0.0133	47.0	0.0199	4.0	0.0014	25.7	0.0070	47.0	0.0090
17.7	0.0052	46.3	0.0079	2.0	0.0003	20.7	0.0058	33.7	0.0051
27.7	0.0038	62.7	0.0130	3.7	0.0003	17.7	0.0063	31.3	0.0045
7.7	0.0162	58.7	0.0284	7.7	0.0008	29.3	0.0191	28.0	0.0122

HM027		HM028		HM101	
Number	Mass	Number	Mass	Number	Mass
31.7	0.0007	24.7	0.0037	19.3	0.0005
35.7	0.0106	28.7	0.0093	35.0	0.0090
31.0	0.0090	48.7	0.0162	82.7	0.0091
60.0	0.0087	49.3	0.0063	91.7	0.0083
54.7	0.0119	64.7	0.0145	65.0	0.0085
37.7	0.0086	41.0	0.0143	36.7	0.0072
44.3	0.0100	59.0	0.0168	58.0	0.0101
42.7	0.0086	52.3	0.0149	56.7	0.0090
25.0	0.0083	58.7	0.0186	44.3	0.0077
45.7	0.0130	62.3	0.0147	46.7	0.0139
42.3	0.0082	48.3	0.0111	63.3	0.0080
68.7	0.0115	49.7	0.0115	118.7	0.0133
35.3	0.0117	66.7	0.0159	69.0	0.0145
40.0	0.0164	64.7	0.0183	35.0	0.0112
54.3	0.0115	53.7	0.0153	25.7	0.0090
41.7	0.0123	47.3	0.0142	78.0	0.0166
29.0	0.0096	58.3	0.0167	88.3	0.0105
50.7	0.0172	41.3	0.0135	76.3	0.0174
23.0	0.0048	45.3	0.0176	111.7	0.0141
57.7	0.0145	91.7	0.0213	100.0	0.0083
60.3	0.0168	58.7	0.0151	42.0	0.0093
25.3	0.0118	56.3	0.0209	74.0	0.0127
28.7	0.0088	59.0	0.0173	33.7	0.0173
36.0	0.0105	78.3	0.0162	61.0	0.0135
63.3	0.0032	71.0	0.0060	61.0	0.0037
27.3	0.0040	24.3	0.0028	58.3	0.0041
0.0	0.0000	0.0	0.0000	0.0	0.0000
36.0	0.0080	45.7	0.0157	38.0	0.0071
34.3	0.0147	56.0	0.0138	66.0	0.0165
38.7	0.0111	82.3	0.0200	39.0	0.0082
47.0	0.0137	52.7	0.0182	47.7	0.0171
21.0	0.0078	55.0	0.0202	37.7	0.0094
29.7	0.0013	5.7	0.0019	35.7	0.0122
72.3	0.0065	70.3	0.0086	65.0	0.0078

74.3	0.0038	95.7	0.0041	44.7	0.0030
50.3	0.0039	42.0	0.0046	29.7	0.0026
26.7	0.0019	36.7	0.0043	24.0	0.0019
26.3	0.0063	35.3	0.0120	24.7	0.0107
65.7	0.0046	82.7	0.0064	40.7	0.0022
35.7	0.0037	28.0	0.0089	26.0	0.0031
39.0	0.0038	41.7	0.0121	31.7	0.0049
50.3	0.0037	77.7	0.0091	58.7	0.0119
46.7	0.0086	70.3	0.0164	64.3	0.0126
0.0	0.0000	0.0	0.0000	35.3	0.0022
30.3	0.0060	34.3	0.0135	48.3	0.0029
30.7	0.0101	53.3	0.0135	78.3	0.0190
