# Supplementary material for: Comparative genomics of the core and accessory genomes of 48 Sinorhizobium strains comprising five genospecies
Source: Genome Biol. 2013 Feb 20;14(2):R17. doi: 10.1186/gb-2013-14-2-r17 (PMC4053727; doi:10.1186/gb-2013-14-2-r17)
Supplement: Additional file 2 — Figure S1 and S2. [file gb-2013-14-2-r17-S2.PDF]

Supplemental Figure 1

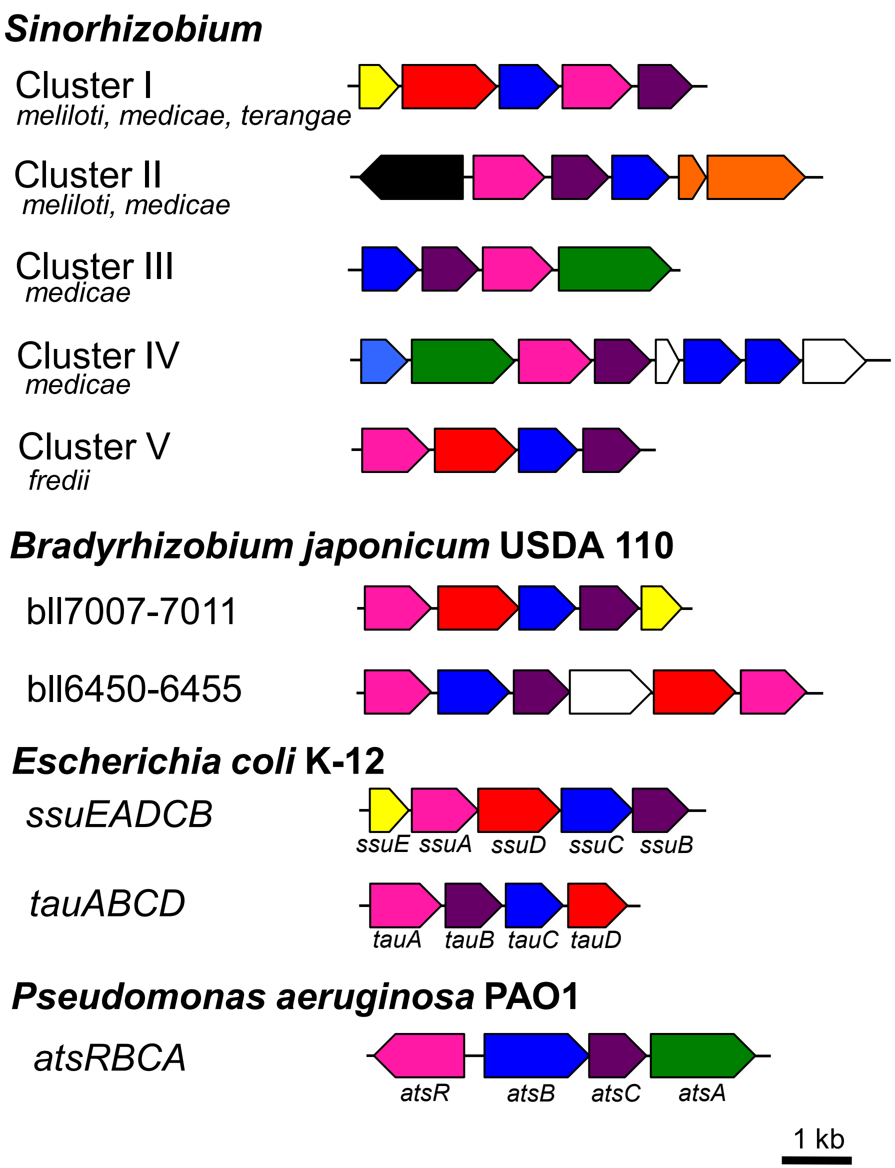

Genetic organization of organic sulfur compounds utilization genes in *Sinorhizobium* strains and their corresponding genes in *Bradyrhizobium japonicum*, *Escherichia coli* and *Pseudomonas aeruginosa*. Clusters containing gene coding for alkane sulfonate monooxygenase ( ), arylsulfatase ( ), taurine dehydrogenase ( ), FMN reductase ( ), ABC transporter periplasmic binding protein ( ), ABC transporter ATP-binding protein ( ), ATP transporter permease protein ( ), and transcriptional regulator ( ). The genes in each gene cluster are orthologs of Smed_4212-4216 (Cluster I), Smed_4858-4863 (Cluster II), Smed_1127-1130 (Cluster III), Smed_3146-3151 (Cluster IV) in *S. medicae* WSM419, and U205v1_2470004-2470007 (Cluster V) in *S. fredii* USDA 205.

Supplemental Figure 2

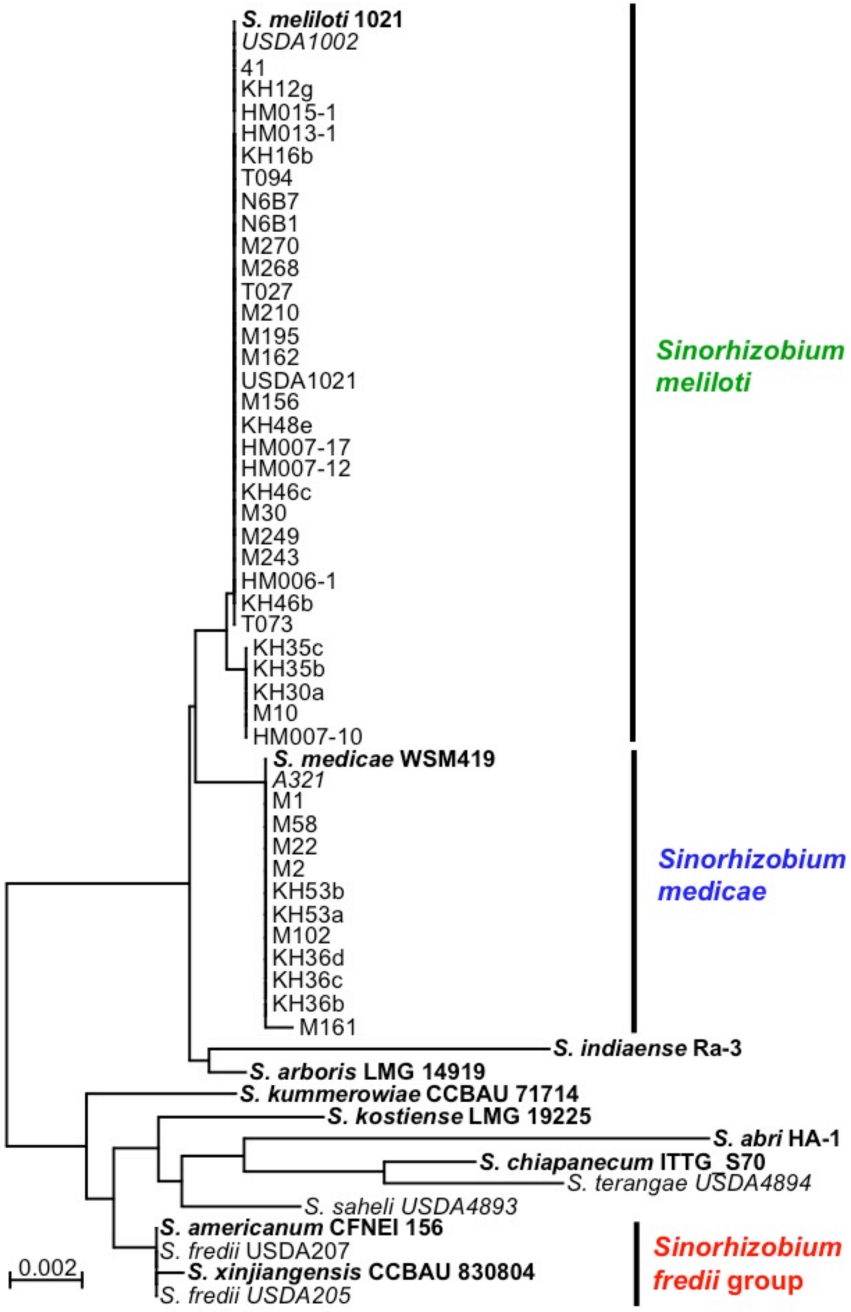

Neighbor-joining tree based on the 16s rRNA gene sequences for the *Sinorhizobium* genus. The strains sequenced in this study were in non-bold font. The reference strains were in bold font and the type strains were in italic font. Bar indicates number of substitutions per site. The reference strains used for this analysis were *S. meliloti* 1021 (accession no. NC_003047.1), *S. medicae* WSM419 (NC_009636.1), *S. indiaense* Ra-3 (AB015420.1), *S. arboris* LMG 14919 (AM181744.1), *S. kummerowiae* CCBAU 71714 (NR_042720), *S. kostiense* LMG 19225 (NR_042484.1), *S. abri* HA-1 (AB015421), *S. chiapanecum* ITTG_S70 (EU286550), *S. americanum* CFNEI 156 (NR_025251), and *S. xinjiangensis* CCBAU 830804 (AF250355).
